# Process Simulation and Comparative Techno-Economic Assessment of Fish Oil Recovery from Fish Heads via Supercritical CO_2_ Extraction and Enzymatic Hydrolysis

**DOI:** 10.3390/foods15183183

**Published:** 2026-09-09

**Authors:** Gebremichael Gebremedhin Hailu, Anand Kumar, Sharmin Zaman Emon, Syed Mohammad Ehsanur Rahman, Zefu Wang, Yang Liu, Shuai Wei, Shucheng Liu

**Affiliations:** 1 College of Food Science and Technology, Guangdong Ocean University, Guangdong Provincial Key Laboratory of Aquatic Products Processing and Safety, Guangdong Province Engineering Laboratory for Marine Biological Products, Guangdong Provincial Engineering Technology Research Center of Seafood, Key Laboratory of Advanced Processing of Aquatic Product of Guangdong Higher Education Institution, Zhanjiang 524088, China; 2 Department of Food Engineering, Oda Bultum University, Chiro 226, Ethiopia; 3 Centre for Advanced Research in Sciences (CARS), University of Dhaka, Dhaka 1000, Bangladesh; 4 Department of Animal Science, Bangladesh Agricultural University, Mymensingh 2202, Bangladesh; 5 Collaborative Innovation Centre of Seafood Deep Processing, Dalian Polytechnic University, Dalian 116034, China

**Keywords:** supercritical fluid extraction, enzymatic hydrolysis, process simulation, technoeconomic analysis, fish head oil, SuperPro Designer

## Abstract

The valorization of fish processing by-products, particularly fish heads, represents an opportunity to advance the circular economy and reduce the environmental burden associated with organic waste. This study presents a process simulation-based techno-economic assessment of industrial-scale oil extraction from fish heads using supercritical fluid extraction (SCFE) and enzymatic hydrolysis extraction (EHE). Process models were developed in SuperPro Designer (V9.0) for a 50,000 kg/batch facility, integrating mass and energy balances, equipment design, scheduling, and economic analysis. All the results are derived from process simulations and have not been validated experimentally. SCFE yields a relatively high annual oil output (43.03 million kg/year) but is constrained by high solvent costs. EHE produces less oil (34.06 million kg/year) yet shows superior economic indicators, including a higher return on investment (24.77%), a shorter payback period (4.04 years), and diversified co-product revenues (cake, lecithin, soapstock), although it requires relatively greater capital ($862.7 million compared with $846.2 million for SCFE). While a full life-cycle assessment was not conducted, a partial resource inventory assessment indicates that EHE enables a biorefinery approach that improves resource efficiency. A sensitivity analysis revealed that the most favorable batch capacities were 50,000 kg for the SCFE and 150,000 kg for the EHE under the assumptions and throughput range examined. Overall, this simulation-based study underscores the economic and resource-efficiency potential of integrated biorefinery strategies for fish waste valorization, while highlighting the need for experimental validation and comprehensive environmental evaluation in future work.

## 1. Introduction

Fish and fish byproducts are high in nutrients and are the primary natural sources of omega-3 polyunsaturated fatty acids, particularly eicosapentaenoic acid (EPA) and docosahexaenoic acid (DHA), both of which are highly valued in the food and pharmaceutical industries. Fish byproducts are often discarded as process byproducts, but they are occasionally used as animal feed and fertilizer [1]. In addition to these applications, fish processing by-products, including fish heads, skins, bones, and viscera, have attracted increasing attention as renewable feedstocks for the production of high-value products such as protein hydrolysates, collagen, gelatin, and bioactive peptides [2,3]. The valorization of these by-products through enzymatic hydrolysis, for instance, enables the simultaneous recovery of multiple value-added streams, including protein hydrolysates, collagen, and fish oil, maximizing resource utilization and reducing organic waste disposal [4]. Furthermore, fish oil recovered from by-products has been explored as a suitable feedstock for biodiesel production, offering a sustainable alternative to conventional edible and non-edible oil sources [5]. Further processing of fish by-products to recover high value-added products is significant in two ways. (a) Decreasing environmental pollution and (b) increasing assets for the fishing industry [6]. Fish byproducts are rich in oil (ranging from 1.4% to 40.10%), depending on the species, tissue type, and extraction method used [2].

Due to the numerous disadvantages and inefficiencies of traditional oil extraction, innovative oil extraction methods such as enzyme hydrolysis, supercritical fluid extraction, and ultrasonic extraction have emerged [7]. Conventional lipid extraction methods, including solvent extraction, steam distillation, and mechanical pressing, have several inherent drawbacks. Solvent-based techniques require large volumes of organic solvents, leaving toxic residues and raising significant environmental concerns [8,9]. Thermal methods such as steam distillation and high-temperature cooking are energy-intensive and often cause thermal degradation of heat-labile bioactive compounds, particularly omega-3 polyunsaturated fatty acids [4,10]. Mechanical pressing typically yields low extraction efficiencies, and both conventional approaches involve lengthy processing times and elevated energy demands [5]. These limitations drive researchers to explore greener, more efficient alternatives such as supercritical fluid extraction (SCFE) and enzymatic hydrolysis (EHE), which operate under mild conditions, minimize solvent use, and better preserve sensitive bioactive lipids.

SCFE is a popular green separation process in the food, nutraceutical, and pharmaceutical industries. SCFE allows for the selective extraction of desirable components without leaving hazardous residues in the extracts, lowering the danger of degrading labile chemicals [8]. Enzymatic hydrolysis extracts fish oil by breaking down proteins in samples to release the oil, which is then separated by centrifugation. This process is commonly used to extract fish oil while creating hydrolyzed proteins [11]. Process simulation is a critical tool for evaluating the technical and economic feasibility of new technologies prior to commercialization. It allows for the systematic assessment of mass and energy balances, equipment sizing, resource requirements, and the overall economic performance of a proposed facility [12,13,14].

Although many novel technologies, products, and strategies have been discovered in the laboratory and effectively piloted, scaling them through extension, adoption, and replication has presented significant challenges due to ineffective design relative to raw material characteristics, utility concerns, supply challenges, and economic factors [15]. Process simulation supports system optimization by increasing energy and resource utilization efficiencies, illustrating raw materials, labor, and power supply needs, identifying primary products, and recycling utilities [16]; thus, economic feasibility studies are essential before scaling up.

**Figure 1:**
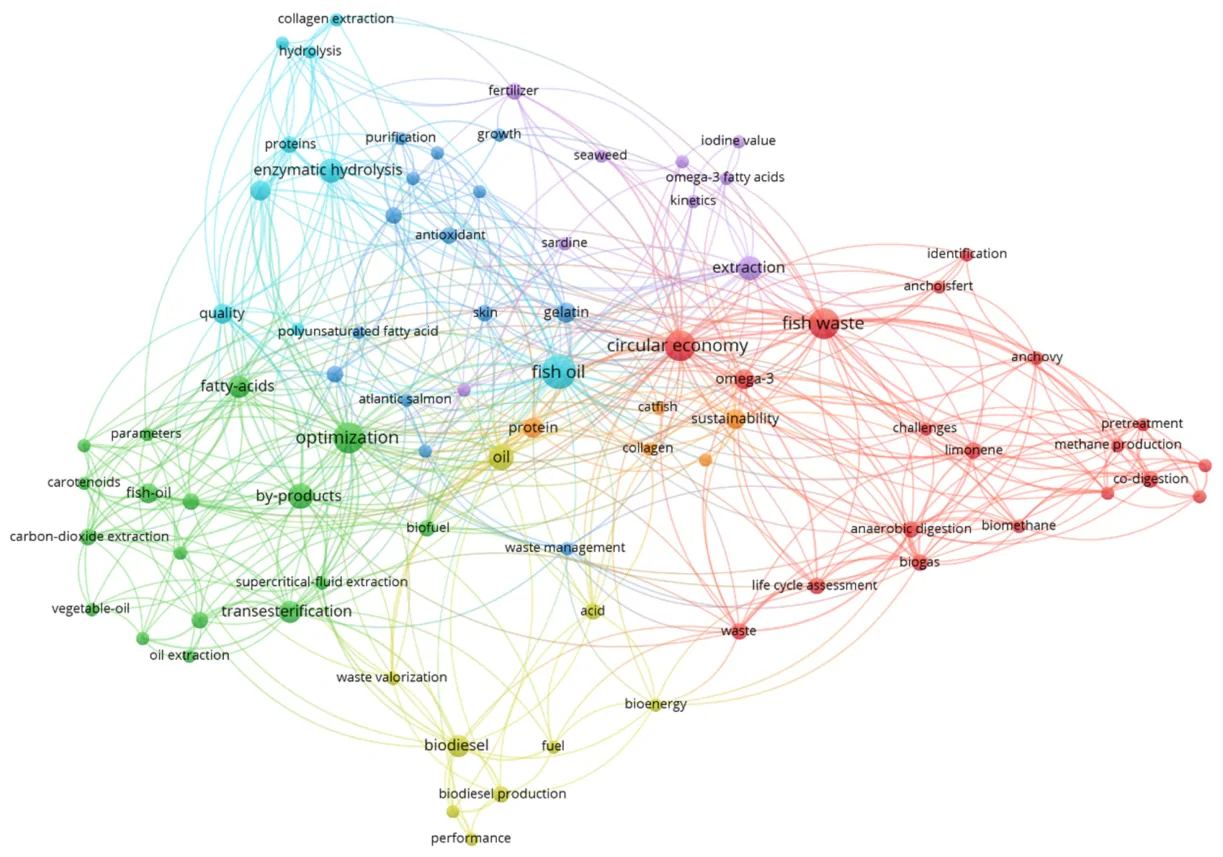
Bibliometric network of keyword co-occurrence in research on fish by-product valorization, extraction, and process development based on the Web of Science literature dataset (2020–2026).

To frame the present study within the broader research landscape and identify knowledge gaps, a bibliometric analysis of the Web of Science Core Collection was undertaken. The literature search was performed on 5 January 2026 using the following query: ((“fish byproducts” OR “fish waste” OR “fish head” OR “fishery byproducts” OR “fish processing waste”) AND (“oil extraction” OR “fish oil” OR “supercritical fluid extraction” OR “enzymatic hydrolysis” OR “oil recovery” OR “technoeconomic analysis” OR “techno-economic analysis” OR “process simulation” OR “SuperPro Designer” OR “scale-up” OR “scale up” OR “circular economy”)). The search retrieved 146 documents. The analysis was restricted to English-language publications, and review articles were excluded. The resulting dataset was analyzed for keyword co-occurrence, thematic clustering, and research trends using VOSviewer (V1.6.2, The Netherlands). A minimum keyword occurrence threshold of two was applied. Of the 378 identified keywords, 82 met the threshold and were included in the final analysis. The results revealed that previous research has focused heavily on fish waste valorization, fish oil recovery, extraction technologies (enzymatic hydrolysis and supercritical fluid extraction), and circular economy approaches. However, process simulation, technoeconomic analysis, and SuperPro Designer did not emerge as dominant keywords, indicating a potential research gap in the integration of extraction technologies with process simulation and techno-economic assessment (TEA) (Figure 1).

The selection of SCFE and EHE for this comparative study is deliberate and rooted in their fundamentally different extraction mechanisms. SCFE is a typical example of a green, solvent-based technology that offers high selectivity and produces a high-purity, solvent-free extract, as demonstrated in recent techno-economic studies on oilseeds [8]. Conversely, EHE represents a biochemical approach that enables the simultaneous production of multiple high-value products, including protein hydrolysates, collagen, and oil, from a single fish waste feedstock, aligning with biorefinery and circular economy principles [4].

The two simulated extraction processes operate under different system boundaries due to the inherent nature of each technology. SCFE is a more direct route for oil recovery, as supercritical ${CO}_{2}$ can be tuned for high selectivity toward non-polar lipids, and systems often employ fractional separation to enhance the purity of the final extract, yielding a high-purity product that requires minimal post-processing. Studies have also demonstrated that, compared with conventional methods, SCFE can effectively reduce impurities and toxic elements [17]. In contrast, the EHE produces a crude oil that is intimately mixed with the reaction media and residual biomass. To achieve a comparable final product quality, specifically a refined, food-grade fish oil suitable for high-value applications, the EHE process inherently necessitates an integrated refining sequence, including degumming, neutralization, bleaching, and deodorization [18]. The presence of free fatty acids, phospholipids, and other impurities in the EHE crude oil makes these refining steps essential for achieving the purity and stability required for market applications [5,18]. Therefore, to ensure a fair techno-economic comparison, both processes are simulated to produce a final refined fish oil meeting minimum and equivalent quality standards. The EHE system boundary inherently includes the refining section as an integral part of its production pathway. For SCFE, while some purification steps such as deodorization can be applied after extraction depending on the target application and final product specifications, the process configuration is considerably less complex due to the high purity of its primary extract.

While process simulation and TEA have been applied to SCFE for other feedstocks such as baru seeds [8] and patchouli [19], and to enzyme-assisted extraction (EAE) for soybean oil [20], these studies focus on conventional oilseeds and do not address the valorization challenges of fish processing by-products. Moreover, none have provided a direct comparative TEA of SCFE versus EHE for the same feedstock. Although several researchers [1,21,22] have compared extraction methods in terms of oil yield, to our knowledge, no published study has yet provided a comprehensive TEA based on raw material cost, processing time, labor, utilities, and yield for oil extraction from fish by-products, specifically fish heads. Therefore, the goal of this research is to simulate and evaluate the technoeconomics of producing oil from fish heads on an industrial scale using SCFE and EHE, employing SuperPro Designer (V9.0, Intelligen, USA). By integrating process simulation, economic evaluation, sensitivity analysis, and batch scheduling, this work aims to provide a scalable and economically informed roadmap for the fishery industry. The present study addresses this gap and supports the valorization of fish head waste as a viable source of oil.

## 2. Materials and Methods

### 2.1. Feedstock Characterization

The composition of the raw fish head waste was obtained from previous works, which reported that it has approximately 65% moisture, 25% oil, and 10% non-oil solids (including proteins, minerals, and carbohydrates) [1,23,24,25,26]. For a batch production system with a 50,000 kg processing capacity, this corresponds to approximately 32,500 kg of water, 12,500 kg of oil, and 5000 kg of non-oil solid content.

### 2.2. SCFE Method

#### 2.2.1. Process Description

**Figure 2:**
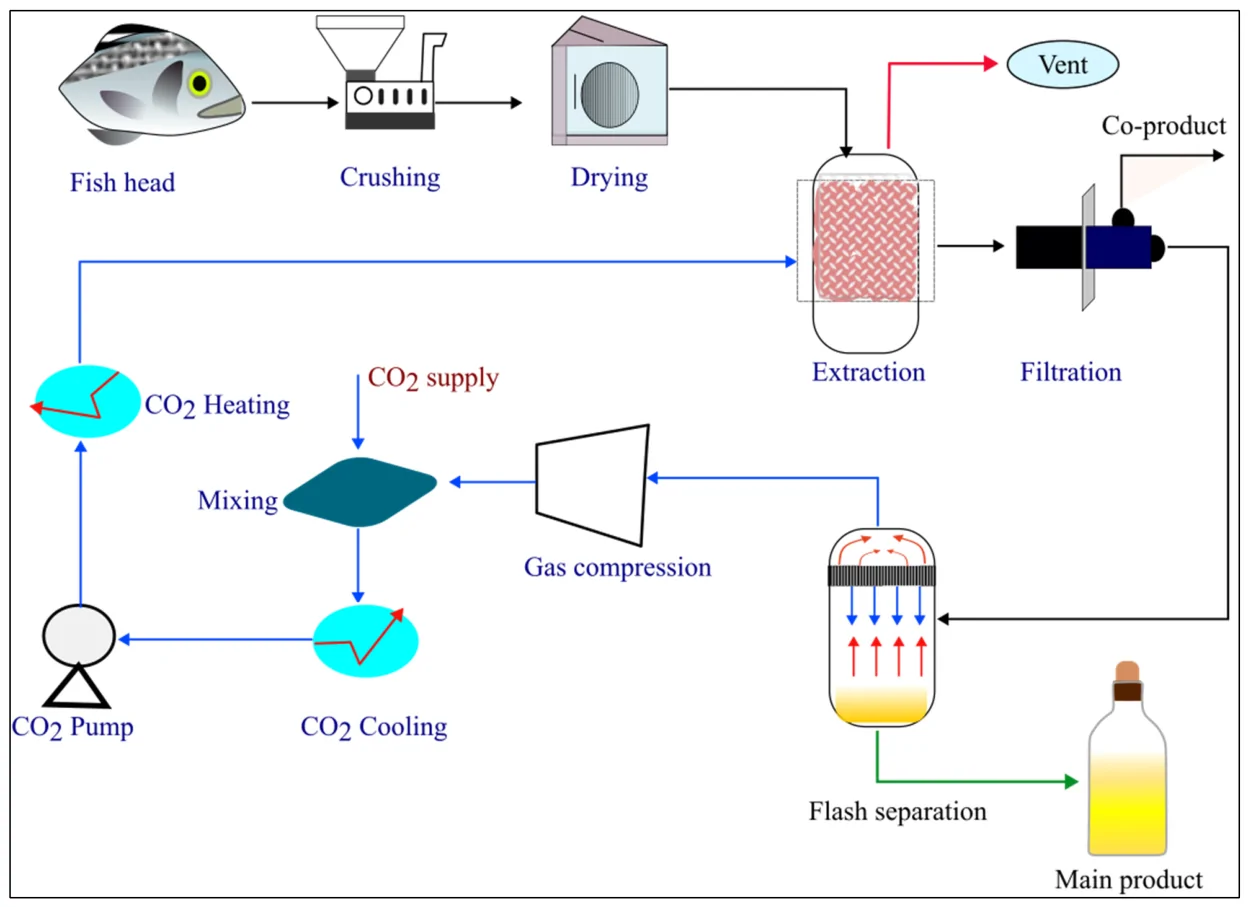
Schematic of the SCFE process for fish head oil recovery. The process comprises raw material pretreatment (milling and drying), the supercritical extraction stage, and the downstream fractionation stage, which includes solvent recycling and recovery of refined fish head oil.

SCFE is conducted through three sequential operational stages: pretreatment, extraction, and fractional separation (Figure 2) [10]. The initial pretreatment stage involves the conditioning of raw sample material, which may include milling to a specified particle size and drying to remove residual moisture, to ensure that the solid matrix is optimally prepared for extraction. During the core extraction stage, the conditioned material is loaded into an extraction column. A supercritical solvent, maintained at temperatures and pressures above its critical point, is then passed through the column, where it solubilizes the target compounds. The resulting solution of solvent and dissolved solute is subsequently conveyed to a separation vessel. Fractional separation is achieved within this vessel by strategically altering the thermodynamic conditions of the system, typically through a reduction in pressure, an increase in temperature, or a combination of both. This manipulation induces a precipitous decrease in the solvent density and solvating capacity, leading to the recovery of the extracted material [27,28]. A standard SCFE apparatus is composed of integrated components that facilitate this process: a chiller and high-pressure pump for solvent conditioning and delivery; an extraction column housed within a temperature-controlled oven; one or more separators for product collection; heat exchangers for thermal regulation of process streams; and a back-pressure regulator to maintain system pressure above the critical point of the solvent.

During the SCFE of oils, the co-extraction of undesired compounds can occur, potentially compromising the quality of the final product. Unlike conventional refining steps such as degumming and bleaching, this process often employs fractional separation, which typically utilizes multiple separators arranged in series to enhance the purity of the extracted oil [29].

#### 2.2.2. Process Setup

##### Upstream Section

**Figure 3:**
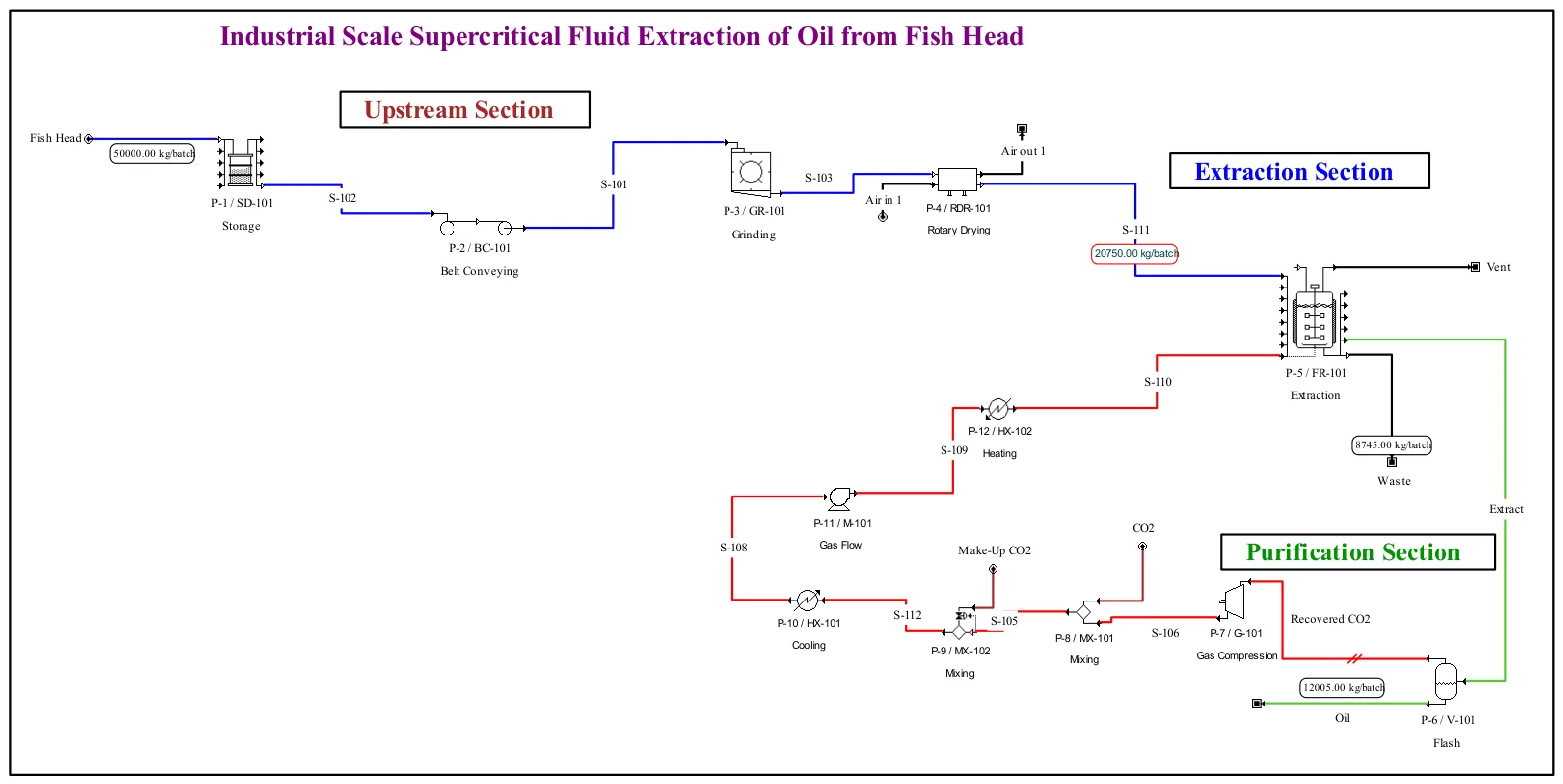
Flow sheeting developed for industrial-scale SCFE of oil from fish head waste.

For this simulation, five pure components were used: ${CO}_{2}$, dry fish head, oil, waste, and water. The exceptions were oxygen and nitrogen, which are registered by default in SuperPro Designer, and the two stock mixtures: the fish head and the default air mixture (Table 1). The fish head $\left(50,000\frac{kg}{batch}\right)$ is fed to a storage tank (P-1/SD-101) and then conveyed (P-2/BC-101) to a grinding section (P-3/GR-101) to reduce the particle size. Following size reduction, the powder is dried in a rotary drier (P-4/RDR-101) for 60 min to a final moisture content of 10% using hot air as a heating medium [21]. While the details of the major operations are presented in Table 2 and the flow sheet is shown in Figure 3, the key unit procedures and their operations are highlighted below.

- Transfer-In-1: Fine powder is fed at a flow rate of 50,000 kg/batch to the rotary dryer.
- Dry-1 (Rotary Drying): The product is dried for 60 min to reduce the moisture content and facilitate extraction. The drying procedure removes 90% of the water from the fish head powder, corresponding to about 29,250 kg/batch and yielding approximately 20,750 kg of dried powder.
- Transfer-Out-1: The dried product is then transported to the subsequent unit operation (extraction) using the transfer-out port (S-111).
- CIP-1 (in-place cleaning): After the first batch is dried and transferred, the dryer is cleaned with water to prevent cross-contamination and improve operational efficiency.

##### Extraction Section

In the extraction step, the dried product is transformed into crude oil and waste (byproduct) in a batch fermenter (P-5/FR-101), which is modeled as a batch stoichiometric reaction. The following unit operations are considered in the extraction procedure.

Transfer-In-1: The dried product is supplied into the fermenter using port S-104 (Figure 3).

Charge-1: Supercritical ${CO}_{2}$ (S-110) is supplied to the extraction reactor at a solvent-to-feed (S/F) mass ratio of 14.5, corresponding to a flow rate of 300,000 kg per batch, based on a dried fish head powder feed of 20,750 kg/batch.

React-1: Multiple unit reactors (P-5/FR-101), with stagged mode set 1, were used to extract oil from dried fish head powder, and the reaction was a stoichiometric reaction. The reactor was set to 45 °C, and a reaction time of 2 h was considered [30]. A portion of ${CO}_{2}$ (5%) is emitted through the vent port, while the remainder is recovered for the next batch. A 98% reaction extent was considered for this reaction. Following 90% water removal, the dried powder composition (on a dry basis, excluding moisture) was calculated as 71.43% oil and 28.57% non-oil solids. These non-oil solids are represented as waste. Based on a target extraction efficiency of 98%, the stoichiometric equation was formulated as presented in Equation (1).

(1) 100 Dried Fish Head=70 Oil+30 Waste

Cool-1: Once the extraction is completed, the extraction system is cooled to 25 °C using glycol as a cooling agent.

Split-1: In this unit operation, ${CO}_{2}$ and oil are split from the extraction vessel and discharged as extracts.

Transfer-Out-1: The extraction byproduct is discharged at a flow rate of 8745 $\frac{kg}{batch}$.

CIP-1 (In-Place-Cleaning): Following the successful completion of the splitting and transfer-out unit operations, the reactor is cleaned with water as the cleaning agent.

##### Downstream Stream Section

In the downstream section, the main product (oil) is separated from ${CO}_{2}$ using flash distillation, and ${CO}_{2}$ is recovered and stored for the next batch. Approximately 95% of the ${CO}_{2}$ is recovered and recycled, whereas the remaining 5% is lost as waste gas vented into the atmosphere.

### 2.3. Enzyme Hydrolysis Method

#### 2.3.1. General Production Descriptions

**Figure 4:**
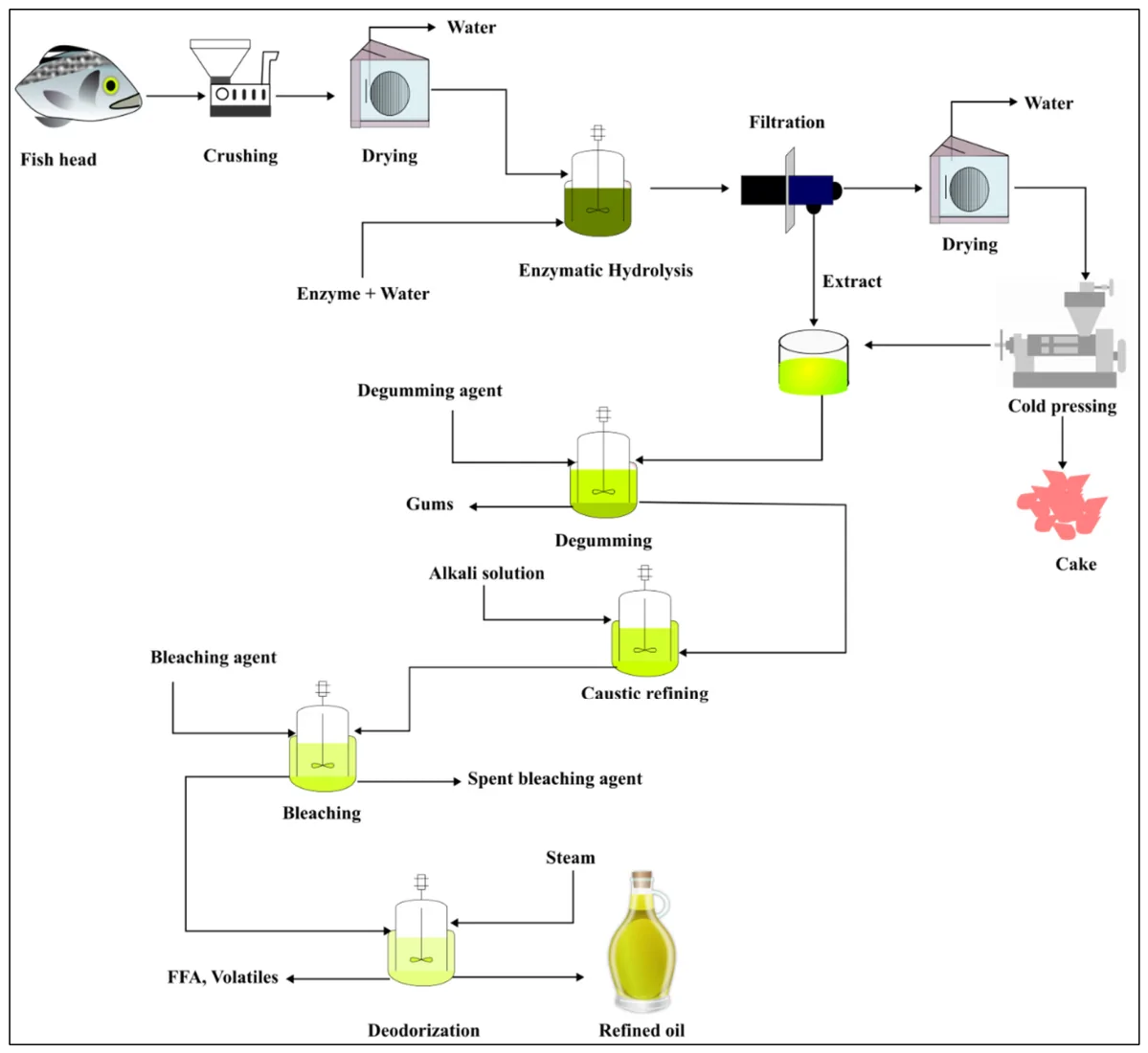
Process flow diagram for the EHE of fish head oil. The integrated process involves pretreatment, enzymatic hydrolysis coupled with cold pressing for oil extraction, and multi-stage refining steps to produce refined fish oil and valuable co-products.

The process of enzymatic hydrolysis for oil extraction (EHE), illustrated in Figure 4, follows the general concept proposed by Cheng, Rosentrater, Sekhon, Wang, Jung and Johnson [20,31], with specific laboratory-scale protocols and optimized parameters adapted from previous work in which some of the authors of the current study participated as co-investigators [1]. The industrial-scale production system was subsequently updated based on other sources cited in this section. The feed is first crushed into small pieces and dried to remove moisture. The dried samples are then subjected to enzymatic hydrolysis in the presence of water and enzymes in a well-equipped reactor. This enzymatic step serves as the primary extraction mechanism, where proteolytic enzymes break down the protein matrix of the fish head tissue, releasing the oil from the cellular structure. Following enzymatic hydrolysis, the released oil is separated, while a portion of the oil remains physically entrapped within the residual solid cake. To maximize overall oil recovery, a cold pressing step is integrated as a complementary mechanical operation to recover the remaining bound oil. This combined approach aligns with findings from similar studies where a wet enzymatic process coupled with pressing significantly reduced residual oil content compared with enzymatic treatment alone [31]. While the integration of enzymatic hydrolysis with cold pressing has been shown to reduce the residual oil content compared to enzymatic treatment alone, the effectiveness of this combined approach is contingent upon factors such as the enzyme cocktail composition, hydrolysis temperature and duration, moisture content, and degree of substrate comminution. The crude oil is then transferred to the purification section, which involves degumming, caustic refining, bleaching, and deodorization.

Degumming removes phosphatides from oil, improving its stability and facilitating further processing. Water degumming can effectively remove the majority of hydratable phosphatides found in crude oil. To further reduce the phosphorus concentration, a version of the water-degumming process known as acid-degumming, which occurs at high temperatures, is utilized [32,33]. Common degumming agents include phosphoric and citric acids. The degummed oil is next refined caustically. The technique of neutralizing or deacidifying degummed oil involves treating it with an aqueous alkali solution. This solution neutralizes residual acid and FFAs, resulting in soaps (also known as soap stock) that adsorb color and precipitate any gums or mucilaginous particles in crude oil. As a result, these undesirable substances are separated in the aqueous phase, which is heavier than the oil phase, using centrifugation [34]. The neutralized, degummed oil is then bleached. The goal of bleaching is to eliminate chlorophyll from the oil, which reduces its color. Notably, every stage of the previous crude oil purification process already involves some degree of color reduction. However, bleaching is required at this point because, in addition to lowering color, it also eliminates leftover soaps, phospholipids, and oxidizing substances. The three processes used in the bleaching process include mixing the oil with the appropriate amount of bleaching chemical, heating the mixture to a bleaching temperature, and then cooling and filtering. Carbon, natural clays, artificial silicates, and activated earth are a few efficient agents for bleaching edible oil [35]. The last step of oil refining, deodorization, involves a steam stripping process in which steam is injected with bleached oil under low absolute pressure (approximately 4 mbar) and high temperature (>230 °C) to vaporize FFAs and odorous compounds, removing them from nonvolatile triglycerides. The deodorized oil has a bland taste, is colorless (as white as water), and contains less than 3% FFAs, making it suitable for consumer usage [34].

#### 2.3.2. Simulation Setup Description

**Figure 5:**
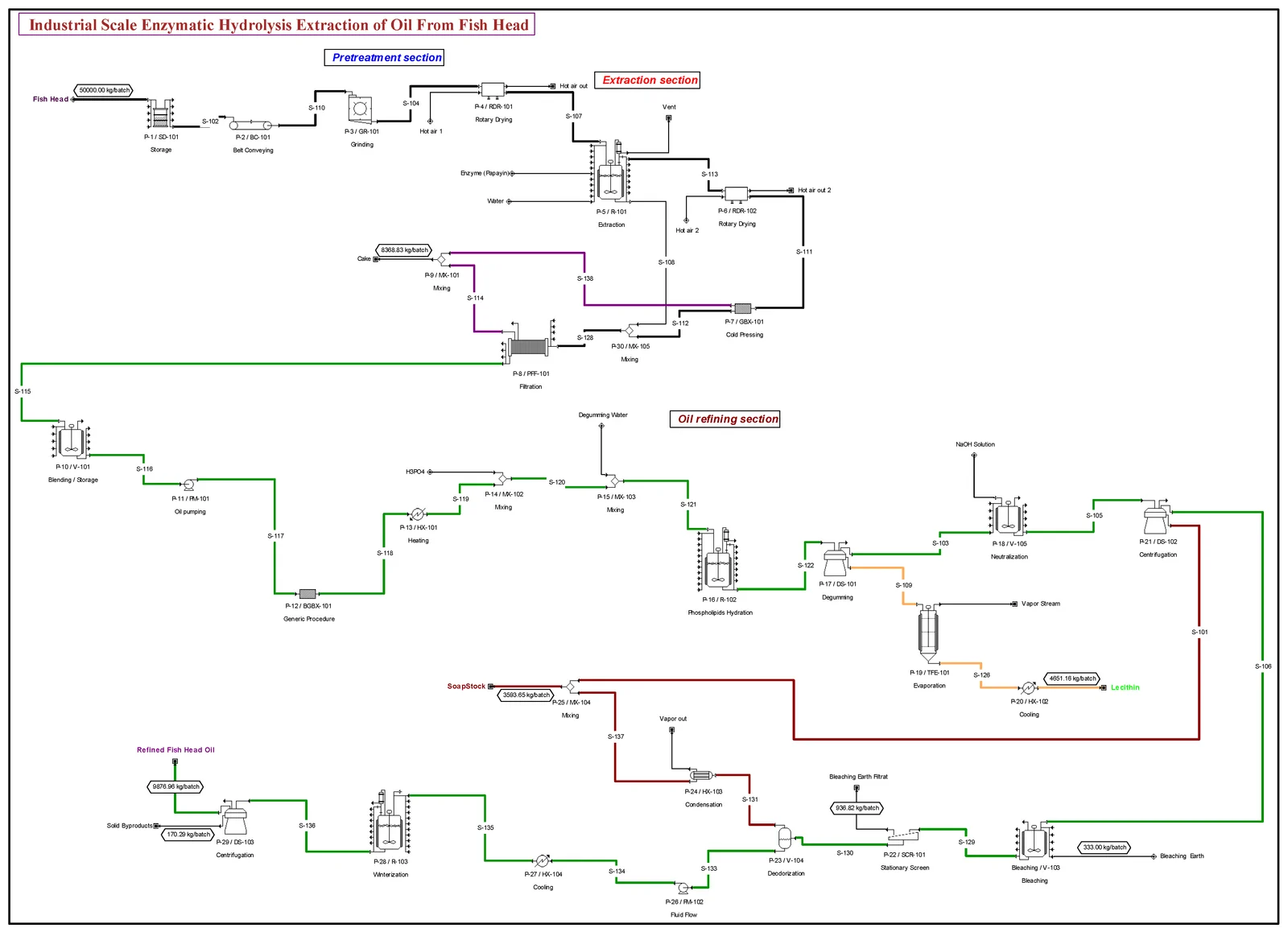
Flow sheeting developed for industrial-scale EHE of oil from fish head waste.

The developed flowsheet for the complete oil extraction process from fish head waste, which employs enzyme-assisted hydrolysis coupled with cold pressing, is presented in Figure 5. The integrated procedure encompasses the sequential stages of raw material preparation, extraction, and oil refining. The components registered both manually and from the SPD Pure Components Data Bank for process simulation are listed in Table 3. In addition, to ensure reproducibility and clarify the process configuration, detailed specifications of the major unit operations and a summary of the process scheduling are provided in Table 4.

##### Raw Material Preparation Section

This section mainly includes raw material reception, conveying, size reduction, and drying. A $\left(50,000\frac{kg}{batch}\right)$ of the fish head is fed to solid storage (P-1/SD-101) and conveyed using a belt conveyor (P-2/BC-101) to the grinding section to reduce the particle size. After the size reduction operation is complete, the raw material is fed to the drying section, in which the moisture content of the material is reduced in a rotary dryer (P-4/RDR-101) for 30 min using hot air as a heating medium.

##### Extraction Section

The oil extraction is completed in two stages, i.e., enzymatic hydrolysis and cold pressing. The dried fish head powder is reacted with water in the presence of papain enzyme in a well-stirred reactor (P-5/R-101) for 3 h. The reaction was considered a stoichiometric reaction, and the balance stoichiometric reaction was as follows. The composition of the raw fish head waste is similar to the values listed in Section 2.1. The overall oil recovery efficiency from the enzymatic step was 85% of the oil present in the dried feed, based on reports from recent studies on fish-head enzymatic hydrolysis [1,36,37]. In addition, approximately 0.5 wt % of dry solids are converted to glycerol due to partial lipase activity accompanying protease action [4]. As a result, the stoichiometric equation, normalized to 100 kg of dried powder entering the extraction column, is expressed as Equation (2).

(2) $$100\;Fish\;Head\overset{Water+Papain}{\to }63.8\;Crude\;Oil+0.45\;Glycerol+35.75\;Cake$$

The extent of the reaction was set to 98%, and the fish head was considered the reaction-limiting reactant. Following the completion of the reaction, a component-based “Split” operation (SPLIT-1) in SuperPro Designer is used to mathematically partition the reaction effluent into two streams, i.e., a crude oil containing glycerol and a cake containing water. In practice, separating the oil from the aqueous phase would require a physical unit operation, such as a three-phase centrifuge; however, this modeling simplification is adopted for the comparative TEA and results in an optimistic scenario for the EHE process. The cake containing water was then transferred to a rotary dryer (P-6/RDR-102) to reduce the water content. The semi-dried sample is subsequently sent to a cold-pressing machine (P-7/GBX-101), and 10% of the cake is considered to be oil. Following cold pressing, the extracted crude oil is mixed with crude oil extracted using enzymatic hydrolysis in a mixing operation (P-30/MX-105) and transferred to a filtration section (P-8/PFF-101) to remove a small fraction of cake that flows with the crude oil. The process time was set to 1 h, and 100% removal of cake from the crude oil was considered. CIP with water as a cleaning agent is continued following filtration to improve the filtration process and avoid cross-contamination.

##### Refining Section

For crude oil extracted using enzymatic hydrolysis, to meet the quality standards necessary for both human and animal consumption, a purification procedure is necessary [38]. First, the crude oil discharged from the filtration section is fed to a storage tank (P-10/V-101) and pumped (P-11/PM-101) to a generic box (P-12/BGBX-101), which involves a chemical reaction that transforms the crude oil into its components as shown in Equation (3) [33,35].

(3) $$\begin{array}{ll} 100\;Crude\;Oil\to & 53\;TAG\;Polyunsaturated+31\;TAG\;Unsaturated\\ & +\;10.5\;TAG\;Saturated\;Proteins+1.3\;Linoleic\;acid\\ & +\;0.8\;Oleic\;Acid+0.4\;Stearic\;Acid+1.2\;Phospholipids\\ & +\;1\;Wax+0.5\;Volatiles+0.3\;Color \end{array}$$

The extent of the reaction was set to 100%, and the crude oil was then heated at 70 °C for 30 min. Following heating, phosphoric acid (0.03% *w*/*w*) was added, and the mixture was mixed (P-14/MX-102). The mixture is then transferred to another mixer (P-15/MX-103), in which degumming water is added from one side. This phospholipid hydration was modeled as a stoichiometric reaction with a reaction time of 1 h and a reaction temperature of 70 °C. In this reaction, phospholipids are converted to phospholipid solids, and the extent of the reaction is set to 85% [39,40]. The reaction is considered stoichiometric, and the balanced chemical equation is presented below as Equation (4).

(4) 100 Phospholipids→100 Phospholipids Solid

Next, the degummed oil is subjected to centrifugation at P-17/DS-101 to remove the gums, and the degummed oil is sent for neutralization (P-18/V-102), while the gum solids are dewatered in a thin film evaporator (P-19/TFE-101), cooled down in HX-102, and packed as a byproduct called lecithin. In the neutralization reaction, four reactions occur, and a NaOH solution is fed as a neutralizing agent [41,42]. The major objective of this stage is to remove the primary fatty acids (linoleic, oleic, and stearic acids) from the crude oil, as described in Equations (5)–(8).

Reaction 1 (stearic acid saponification):

(5) 40 Sodium hydroxide+284.4 Stearic Acid→306.4 Soaps+18.02 Water

Reaction 2 (oleic acid saponification):

(6) 40 Sodium hydroxide+282.47 Oleic Acid→304.45 Soaps+18.02 Water

Reaction 3 (Linoleic acid saponification):

(7) 40 Sodium hydro+280.45 Linoleic Acid→302.43 Soaps+18.02 Water

Reaction 4 (Phospholipid Hydration):

(8) 147.6 Phospholipids→147.6 Phospholipids Solid

Once the reactions are complete, the contents are transferred to a centrifugation unit, and the soap is removed using a disk stack centrifuge (P-21/DS-102). Another important stage in crude oil refining is the removal of color agents, which is performed in a blending tank (Bleaching-V-103) using bleaching earth at a heating temperature of 90 °C for 30 min. The mixture is then transferred to a stationary screen where bleaching earth, with some impurities and color agents are removed. Finally, the degummed and bleached oil is subjected to flash distillation (P-23/V-104) (210 °C, 1.03 bar) to remove all volatile components in the oil, including free fatty acids. The vapor stream is then condensed in the condenser (P-24/HX-103), sent to the soap stock mixture (P-25/MX-104), and mixed with the previous soap stock discharged from the centrifugation unit operation (P-21/DS-102). The liquid stream of the flash distillation, constituting the final product, is then pumped (P-26/PM-102) to the cooling section (P-27/HX-104) and transferred to a reactor (P-27/R-103) where waxes are removed. The reaction is held for 3 h at a reaction temperature of 12 °C, and the reaction is considered a stoichiometric reaction with a 98% extent of reaction. Similarly to the enzymatic hydrolysis stage, this stage requires a long process time. The design uses multiple reactors to increase the number of batches per year and improve throughput. The conversion of wax to precipitated wax was modeled using the stoichiometry given in Equation (9) [33,43].

(9) 147.6 Wax→147.6 Precipitated/Wax

The precipitated waxes are then removed using centrifugation (P-28/DS-103) and discharged as underflow. Finally, the overflow, which is the main product of the extraction (degummed, bleached, neutralized, deodorized, and dewaxed) oil, is then discharged, packed, and sold as purified oil from the fish head.

### 2.4. Process Scheduling and Debottlenecking

For both extraction methods, the batch durations were based on optimum values reported in the literature [1,9,19,22,44,45,46,47,48] and were adapted from similar large-scale plants operating locally. To clearly understand the scheduling and execution of a batch process, a Gantt chart was generated using the SuperPro Designer simulator. An equipment occupancy chart was also created to indicate the occupancy of different equipment units in a batch operation over time.

### 2.5. Economic Evaluation

Cost analysis reports (i.e., Economic Evaluation Reports (EERs), Cash Flow Analysis Reports (CFRs), and Itemized Cost Reports (ICRs)) are generated from the simulation software. The simulations used US dollars as the currency. The direct fixed capital (DFC), equipment purchase cost (taking into account equipment specifications and sizing), and purchase cost of the fish head waste and other raw materials are mentioned.

### 2.6. Economic Assumptions

All economic evaluations were performed in US dollars. Equipment purchase costs were obtained from the built-in SuperPro Designer equipment cost database, which is derived from vendor quotes and scaling algorithms based on capacity (2026 basis). Installation, piping, instrumentation, and indirect costs were calculated using the software’s default multipliers, which are consistent with chemical process industry standards. The labor rates were set based on the SuperPro Designer adjusted basic rate ($69/h) and lumped rate ($50/h). Details of the major raw materials and products, along with their purchase and selling prices and major utilities, are presented in Table 5.

The economic evaluation assumed a 15-year project lifetime with 30 months of construction and a 4-month startup period. The construction costs were distributed as 30% in Year 1, 40% in Year 2, and 30% in Year 3. Depreciation was calculated using the straight-line method over 10 years with a 5% salvage value. An income tax rate of 40% was applied, and depreciation was subtracted from net profit. The base case discount rate for NPV calculations was 7%, with sensitivity analyses at 9% and 11%. A 4% inflation rate was applied to update equipment costs. The contingency and contractor’s fees were set at 10% and 5% of the total plant cost, respectively. Working capital was estimated at 5% of direct fixed capital, with 0% debt financing assumed in the base case. The facility location was assumed to be Zhanjiang, China, with 100% capacity utilization after startup.

### 2.7. Model Validation and Limitations

The process models presented in this study are based on established thermodynamic principles, stoichiometric relationships, and equipment sizing algorithms implemented in SuperPro Designer (V9.0). Key input parameters, including feedstock composition, extraction efficiency, reaction stoichiometry, and process scheduling, were derived from peer-reviewed literature and are presented in detail in Section 2.1, Section 2.2 and Section 2.3. While the individual unit operation models (e.g., rotary drying, flash separation, and centrifugation) are built on validated engineering correlations, the integrated process models have not been experimentally validated at the pilot or industrial scale. This constitutes a limitation of the present study, and future work should prioritize experimental validation of the complete process chains. The simulation results presented herein are intended to provide a comparative techno-economic framework to guide technology selection and identify key scale-up considerations, rather than to serve as definitive performance predictions.

## 3. Simulation Results and Discussion

### 3.1. Mass Balance, Energy Balance, and Process Efficiency

#### 3.1.1. Mass and Energy Balance

Mass balance closure was verified for both extraction processes on a per-batch basis using the component tracking functionality of SuperPro Designer. For the SCFE process, the total mass input (including fish head feed, 300,000 kg/batch of circulating ${CO}_{2}$ entering the reactor, make-up ${CO}_{2}$, water, and drying air) is balanced with the combined output streams comprising refined oil, solid waste, vent gases, and ${CO}_{2}$ recycled to subsequent batches (Table 6). The circulating ${CO}_{2}$ (300,000 kg/batch) is continuously recycled, with 285,000 kg/batch recovered and returned to the system, whereas the remaining 15,000 kg/batch (5%) represents losses that must be replenished by 14,900 kg/batch of make-up ${CO}_{2}$. Thus, while the actual solvent flow through the extraction system is 300,000 kg/batch (25.0 kg ${CO}_{2}$ per kg of oil produced), the net make-up requirement is only 14,900 kg/batch, corresponding to 53.4 million kg annually and 1.241 kg make-up ${CO}_{2}$ per kg of oil produced. For the EHE process, the total mass input (including fish head feed, water, enzyme, hot air, and other chemicals) is balanced with the combined output streams comprising refined oil, cake, lecithin, soapstock, solid byproducts, and water vapor removed during drying operations.

Based on the 50,000 kg/batch feed and 25% oil content, the oil recovery was 24.01% for SCFE (12,005 kg/batch) and 19.75% for EHE (9876.96 kg/batch), corresponding to 96.04% and 79.02% of the total oil in the feed (12,500 kg), respectively. The higher recovery for SCFE reflects the high selectivity of supercritical ${CO}_{2}$ for non-polar lipids, while the lower recovery for EHE reflects cumulative losses during enzymatic hydrolysis, cold pressing, and subsequent refining steps.

These findings are consistent with literature reports, where SCFE of fish byproducts has achieved oil extraction efficiencies ranging from 90% to 98% depending on operating conditions and feedstock characteristics, while enzymatic hydrolysis combined with cold pressing has been reported to recover 75% to 85% of the oil from fish processing byproducts [1,9,37,47].

Energy balance closure was also verified based on stream enthalpy data calculated by the software for each unit operation. For SCFE, the primary energy demand is associated with ${CO}_{2}$ compression (250 bar) and heating to supercritical conditions, as well as hot air generation for drying. For EHE, the energy demand is dominated by hot air generation for drying (474,804 kg/batch) and steam requirements for heating during enzymatic hydrolysis and refining steps. These enthalpy calculations formed the basis for the utility cost estimates presented in Section 3.2.

#### 3.1.2. Process Efficiency and Material Utilization

The material utilization efficiencies for both extraction methods are summarized in Table 7. The SCFE process results in high oil recovery. While the actual solvent flow through the extraction system is 300,000 kg ${CO}_{2}$ per batch (25.0 kg ${CO}_{2}$ per kg oil produced), the 95% recycle rate reduces the net make-up requirement to only 14,900 kg per batch (1.241 kg make-up ${CO}_{2}$ per kg oil). This high solvent efficiency reflects the excellent mass transfer properties and selectivity of supercritical ${CO}_{2}$ for lipid extraction [56]. However, the process requires substantial energy to maintain supercritical conditions (250 bar) and is supported by high hot air consumption (12.18 kg/kg MP) for drying.

The EHE process, while achieving lower oil recovery (19.75% of the total feed mass, facilitates comprehensive biomass utilization through multiple co-product streams (cake, lecithin, soapstock, and solid byproducts). This exemplifies the biorefinery concept, which is gaining prominence in the sustainable processing literature [9,47]. The process consumes 13.53 kg of water per kg of oil produced, reflecting its dual role as a reaction medium and for post-extraction washing, which is consistent with the aqueous environment necessary for enzymatic processing. The enzyme consumption of only 0.047 kg papain per kg of oil recovered indicates excellent catalyst efficiency [57,58]. With respect to resource utilization, the SCFE process generates a single solid residue stream (waste), whereas the EHE process distributes the non-oil biomass across multiple valuable co-products, maximizing the valorization of the feedstock. The energy intensity, reflected in utility costs, is substantially higher for EHE ($11 million/year) than for SCFE ($5.1 million/year), driven primarily by steam requirements for heating during enzymatic hydrolysis and refining steps. However, the EHE process generates negligible waste requiring disposal, as most of the biomass is recovered as co-products.

### 3.2. Economic Performance

#### 3.2.1. Capital Investment

The EHE process requires a slightly higher total capital investment (approximately $862.7 million) than the SCFE process (about $846.2 million), representing a difference of approximately 2% (Table 8). This is largely attributed to the extensive refining section of the EHE process, which includes multiple centrifuges, reactors, and drying units. This is reflected in the equipment purchase cost alone, i.e., $131.5 million for EHE compared to $35.1 million for SCFE. For SCFE, the capital cost is driven primarily by the need for multiple parallel extraction reactors and high-capacity compressors to maintain the supercritical ${CO}_{2}$ recycle loop at 250 bar. The equipment purchase costs for both processes were derived directly from the built-in cost database of SuperPro Designer, which provides vendor-quoted prices scaled to the required capacities. In actual industrial practice, these costs may be reduced through supplier negotiations, bulk purchase discounts, or material substitutions based on site-specific affordability and availability. Capital intensity may pose a barrier to entry for small-to-medium enterprises, despite the superior economic returns of EHE in the long run.

The balance between the high total capital investment for EHE and its short payback period (4.04 years) is explained by the distinct operating cost structure and revenue profile of the process. Although EHE requires significantly greater upfront investment, facility-dependent costs dominate the annual operating expenses (approximately 56%). These costs are relatively fixed and become diluted as the production volume increases, enabling rapid capital recovery. Additionally, the diversified revenue streams of EHE, including cake, lecithin, and soapstock, contribute approximately 11.5% of total revenue, reducing reliance on oil sales alone and accelerating payback. The lower unit production cost for EHE ($8.01/kg oil) compared to SCFE ($10.62/kg oil) further enhances the gross margin (45.50%) compared to SCFE (20.52%), allowing the high initial outlay to be recovered within a short timeframe despite the capital intensity.

The return on investment (ROI) is calculated by dividing the annual net profit by the total capital investment [59], as expressed in Equation (10). Annual net profit is defined as gross profit minus income taxes plus depreciation Equation (11).

(10) $$ROI=\frac{Net\;profit}{Total\;investment}\times 100$$

(11) Net profit=Gross profit−Taxes+Depreciation

The annual net profit and total capital investment were derived from the Economic Evaluation Report (EER) generated by SuperPro Designer. For SCFE, the annual net profit is approximately $144.8 million, yielding an ROI of 17.11%. For EHE, the annual net profit is approximately $213.7 million, yielding an ROI of 24.77%. The higher ROI for EHE reflects its superior gross margin, diversified revenue streams, and lower unit production cost, which collectively offset the higher capital investment.

#### 3.2.2. Operating Cost Structure

**Figure 6:**
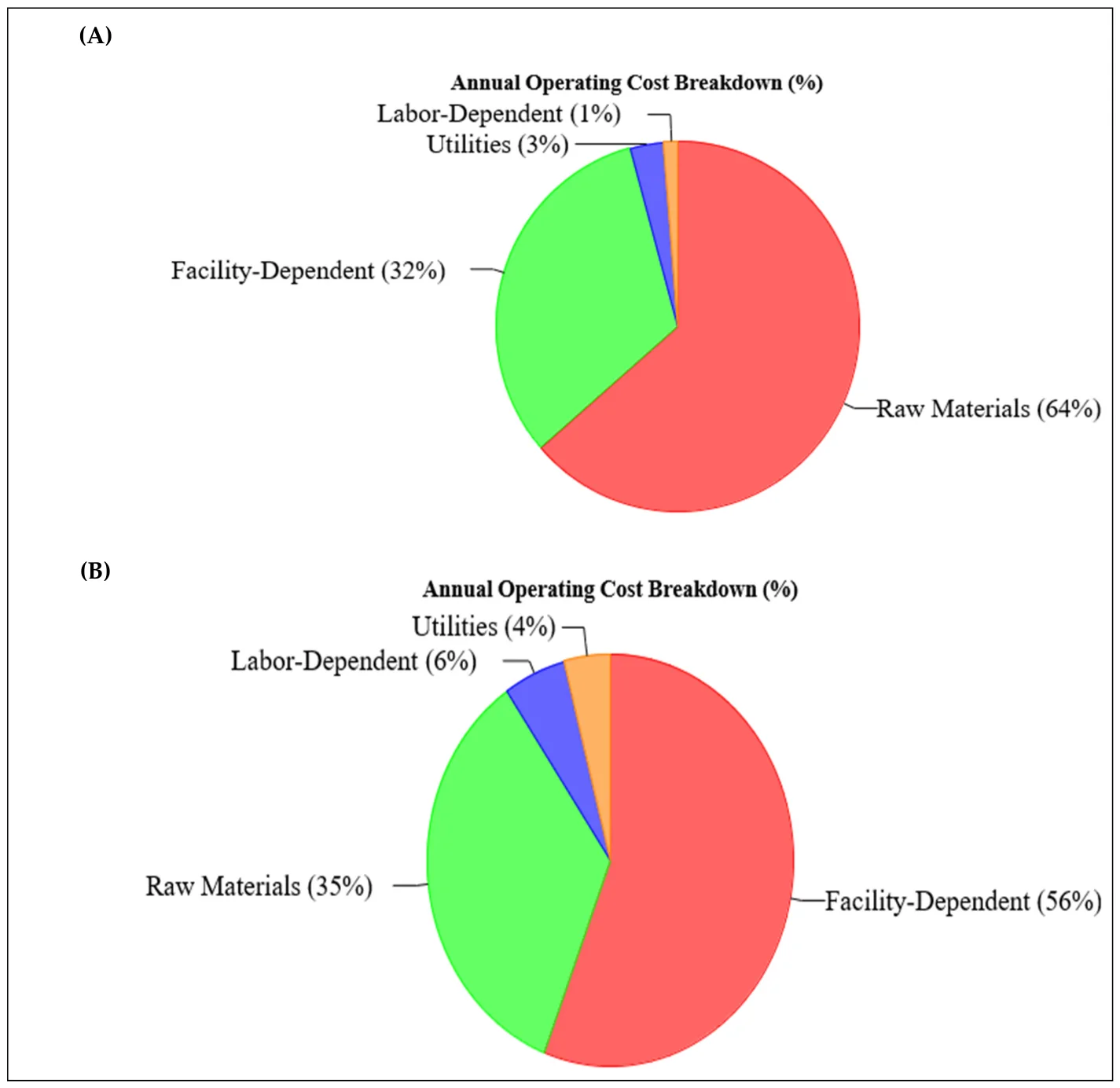
Annual operating cost breakdown; (**A**) SCFE, (**B**) EHE.

A significant difference in the composition of the annual operating cost between the two extraction methods was observed (Figure 6). In SCFE, raw materials, specifically ${CO}_{2}$, constitute 63.63% of operating costs, which underscores the sensitivity of this method to solvent price volatility. Studies note that the cost of ${CO}_{2}$ is subject to variation based on factors such as feedstock sourcing, energy costs, and technological efficiency [60]. Consequently, the economic viability of this extraction method may be highly sensitive to ${CO}_{2}$ price fluctuations. On other hand, the operating cost for EHE is dominated by facility-dependent expenses (55.89%), with raw materials such as water and enzymes accounting for only 34.54% of the total cost. This finding indicates that the EHE method is less prone to raw material price fluctuations but is more dependent on capital recovery and plant utilization.

#### 3.2.3. Revenue Breakdown

The revenue structures of the two extraction methods differ substantially due to their distinct product portfolios. For SCFE, revenue is derived primarily from refined fish oil, which accounts for approximately 98.4% of total revenue ($559.1 million/year), with the remaining 1.6% ($9.2 million/year) coming from the solid byproduct. In contrast, EHE generates revenue from multiple streams. Refined fish oil contributes approximately 88.5% ($443.3 million/year), while co-products, including cake, lecithin, and soapstock, account for the remaining 11.5% ($57.3 million/year). This diversified revenue model makes the EHE less vulnerable to oil price fluctuations and contributes to its superior economic performance despite lower oil yield and higher capital investment.

The refined fish oil obtained from both extraction methods is suitable for high-value applications in the nutraceutical, pharmaceutical, and functional food industries, due to its high content of omega-3 polyunsaturated fatty acids (EPA and DHA) [3,61]. The estimated selling price of refined fish head oil used in this study ($13.00/kg) is based on current market prices for food-grade fish oil. The co-products from the EHE process also hold significant market value; protein-rich cake can be utilized as animal feed or fertilizer, lecithin can be used as an emulsifier in food and pharmaceutical formulations, and soapstock can be processed into animal feed additives or used as a feedstock for biodiesel production [5,62].

#### 3.2.4. Labor and Utilities

**Figure 7:**
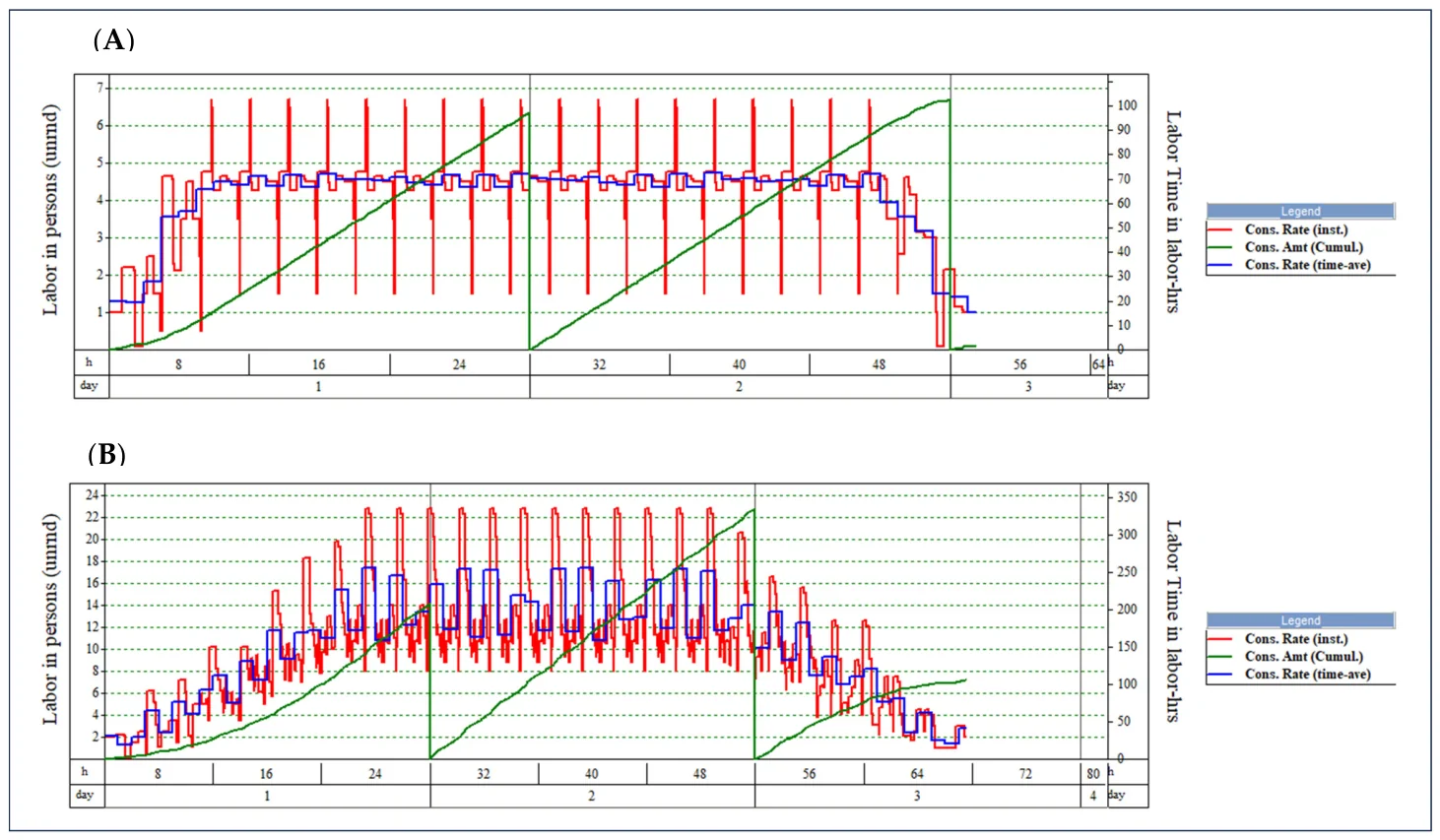
Total labor demand/20 batches of main recipe; (**A**) SCFE, (**B**) EHE.

In industrial process engineering, labor demand profiles serve as critical indicators of operational efficiency, workforce allocation, and economic feasibility [63]. The estimated labor demand for both extraction methods is presented in Figure 7. The graph illustrates three key metrics: Instantaneous Labor Consumption, which depicts a stepwise labor demand over time; the Time-Averaged Labor Consumption Rate (calculated at 1 h intervals), representing a smoothed hourly labor demand; and the Cumulative Labor Consumption (derived from daily time-averaged values), which reflects the total labor-h accumulated over the operational period.

The EHE process demonstrates a highly variable labor pattern, with instantaneous requirements fluctuating sharply from 0.1 to 22.8 persons. These peaks correspond to intensive batch operations such as substrate handling, enzyme dosing, and product recovery, necessitating flexible staffing models, shift rotations, and cross-trained personnel. Over approximately 63.5 h, the EHE process accumulates about 105.96 labor-h, concentrated in sporadic, high-intensity intervals. In contrast, the SCFE process maintains a stable labor profile, with demand consistently ranging between 1.5 and 6.7 persons throughout its 49.5 h duration, reflecting the automated, continuous nature of the process. Although the cumulative labor consumption in the SCFE process of 102.80 h is comparable to that in the enzymatic process, its uniform distribution supports predictable staffing with smaller, fixed crews and reduces the overhead related to scheduling and temporary labor.

The divergent labor dynamics of these two extraction methods illustrate a trade-off between process flexibility and operational stability. Labor variability in the EHE process implies higher indirect costs, including supervision complexity, overtime expenditures, and potential inefficiencies during low-activity phases. The steady labor profile of the SCFE process facilitates more accurate budgeting, lower administrative burden, and enhanced ergonomic conditions due to consistent workload distribution. Ultimately, the analysis underscores that labor demand is not merely an operational detail but a critical design parameter that shapes both the scalability and the sustainability of industrial bioprocesses.

**Figure 8:**
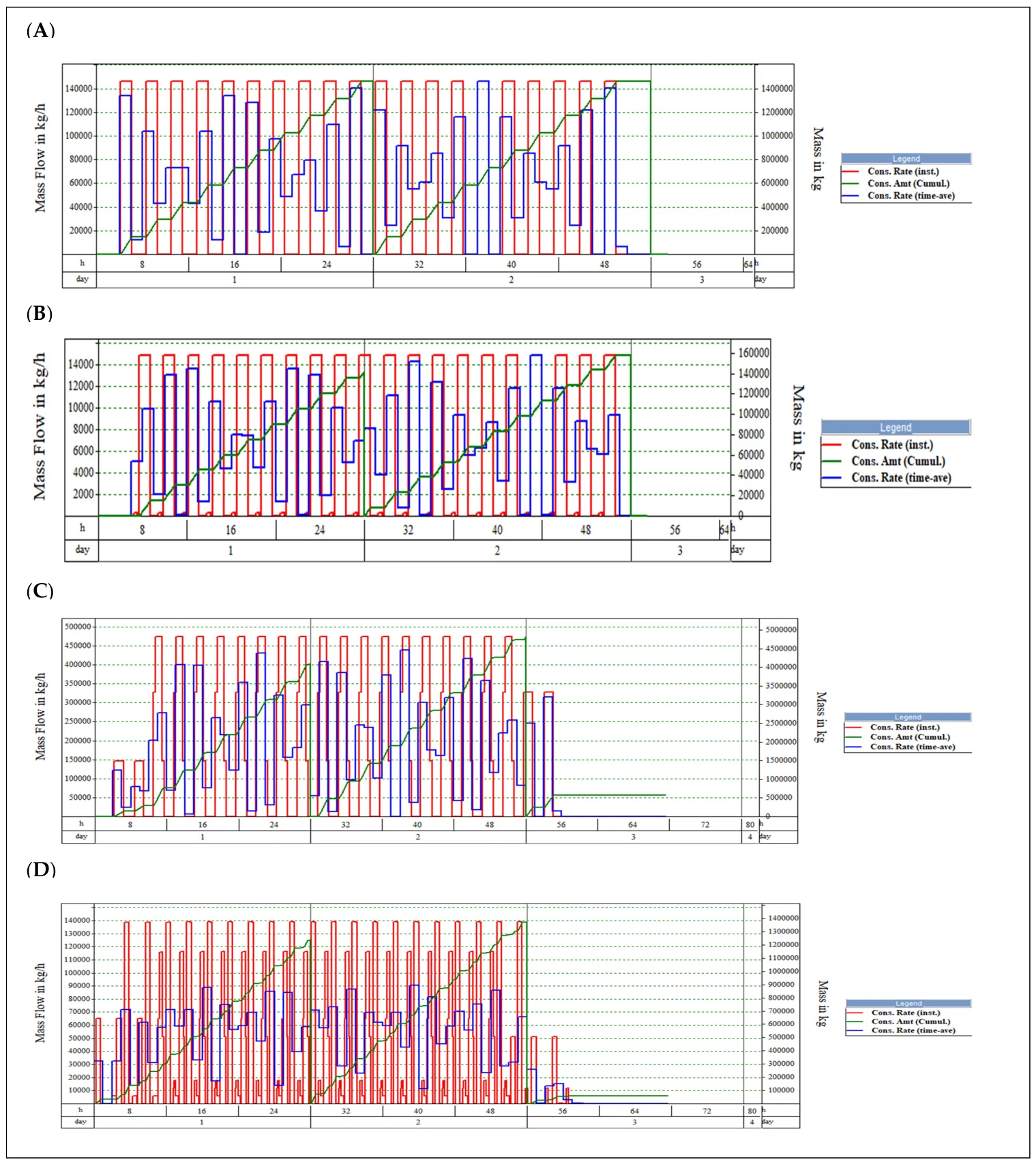
Graphs showing major material demands: (**A**) hot air demand for the SCFE, (**B**) ${CO}_{2}$ demand for the SCFE, (**C**) hot air demand for the EHE, (**D**) water demand for the EHE.

To highlight and compare the utility requirements of the two extraction methods, graphs showing the demands for major materials (i.e., hot air and ${CO}_{2}$ for SCFE, and hot air and water for EHE are presented in Figure 8. A comparative analysis of utility requirements across 20 consecutive production batches reveals significant divergences in the scale, periodicity, and nature of resource consumption between the two extraction methods. These demands are critical for designing and operating industrial-scale plants.

The SCFE method demonstrates a cyclic, pulsed demand for gases. The extraction process circulates a total of 300,000 kg of ${CO}_{2}$ per batch at a solvent-to-feed ratio of 14.5. The consumption pattern shows an instantaneous peak flow of 14,900.00 kg·h^−1^ for make-up ${CO}_{2}$, with a time-averaged maximum of 14,900.00 kg·h^−1^. The cumulative make-up ${CO}_{2}$ consumption is 7903.54 kg per batch, totaling 158,070.83 kg for the 20-batch campaign, reflecting the small fraction of ${CO}_{2}$ lost during the process. The hot air demand for drying the fish head paste peaks instantaneously at 146,249.99 kg·h^−1^, with a cumulative batch consumption of 1,462,500 kg, reaching 29,250,000 kg overall. The relative regularity of these flows suggests a predictable utility load, necessitating robust ${CO}_{2}$ storage, recycling, and compressed air systems.

In contrast, the EHE imposes significantly larger and more dynamic thermal and hydric loads. Hot air consumption reaches an instantaneous peak of 474,800 kg·h^−1^, with a cumulative batch demand of approximately 4.84 million kg. This represents a mass flow rate more than triple that of SCFE, indicating a substantial thermal energy requirement. Water use is highly intermittent, with complex multi-level flow rates peaking at 139,200 kg·h^−1^ and a batch consumption of about 1.37 million kg. This profile necessitates a large buffer capacity, precise flow control, and potentially integrated heat recovery systems. Operationally, SCFE presents a modular, predictable utility profile. The EHE entails greater mass throughput, thermal loads, and dynamic variability in hot air and water. These distinctions influence facility design; i.e., SCFE requires high-pressure ${CO}_{2}$ handling and gas recycling, while EHE demands large-capacity hot air generation, thermal integration, and flexible water management.

#### 3.2.5. Sensitivity Analysis

A systematic sensitivity analysis was conducted to evaluate the economic scalability and parameter sensitivity of both extraction methods. The analysis is divided into two complementary approaches. The first examines throughput sensitivity, which involves evaluating internal scale-up factors controllable by process design. The second assesses multivariable sensitivity, which examines key economic and process parameters that may vary with market conditions, operating context, or process performance.

##### Throughput Sensitivity Analysis

**Figure 9:**
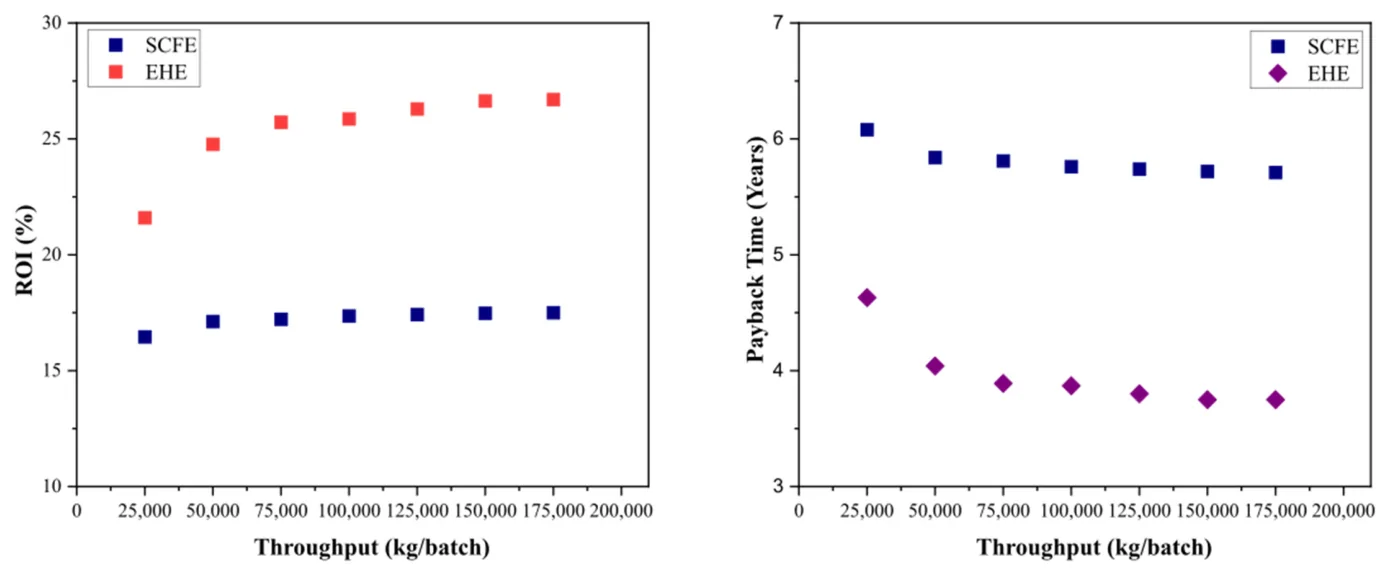
Throughput sensitivity analysis of ROI and payback time for SCFE and EHE as a function of raw fish head feed per batch.

A systematic sensitivity analysis was conducted to evaluate the economic scalability of both extraction methods under varying production scales. Throughput was selected as the independent variable, ranging from 50 to 400% of the base design capacity of 50,000 kg of fish heads per batch. Key performance indicators, including return on investment (ROI) and payback time, were analyzed to delineate the operational and financial boundaries of each technology. The results, summarized in Figure 9, reveal distinct economic trajectories and optimal operating points for the two extraction methods.

The throughput sensitivity analysis for SCFE reveals a strong non-linear response with pronounced diminishing returns. While the absolute ROI increases from 16.46% to 17.50% across the tested range and adapted assumptions, the marginal gain per unit of additional capacity shrinks drastically above the 50,000 kg/batch scale. Scaling from 50,000 to 175,000 kg/batch requires an additional $2.1 billion in capital investment but yields only a 0.38% increase in ROI and reduces payback by a mere 0.13 years. When considering the prohibitive capital intensity and mechanical challenges of ultra-large high-pressure vessels, the practical economic optimum is identified as 50,000 kg/batch. This scale captures the primary economic benefits (improving the ROI by 0.66% and payback by 0.24 years from the 25,000 kg/batch throughput) while avoiding the severe diminishing returns associated with larger capacities.

In contrast, the EHE process exhibits a greater and more linear improvement in financial metrics with increased scale. The ROI rises steadily from 21.60% at 25,000 kg per batch to 24.77% in the base case, and further to 26.64% at 150,000 kg per batch. This robust scalability originates from the capital-intensive nature of EHE, where facility-dependent costs dominate the operating expense structure. Scaling allows for the effective amortization of the high initial investment over a larger production volume. The payback time decreases correspondingly from 4.63 years to an optimum of 3.75 years at 150,000 kg per batch. Although the metrics continue to improve at higher capacities, the rate of enhancement begins to taper beyond this point. Therefore, the optimal throughput for the EHE process is recommended to be 150,000 kg of fish heads per batch. This scale captures the substantive benefits of economies of scale while maintaining operational practicality.

The analysis reveals a strategic dichotomy between the two technologies. SCFE presents a lower capital entry point with a defined and more limited optimal scale, which is suitable for focused production where solvent costs are controlled. The EHE requires greater upfront investment but delivers superior and more scalable financial returns, aligning with large-scale, integrated biorefinery models. This sensitivity assessment provides a clear quantitative framework to guide technology selection based on specific production targets, financial capacity, and long-term strategic goals within the fishery processing industry.

##### Multivariable Sensitivity Analysis

**Figure 10:**
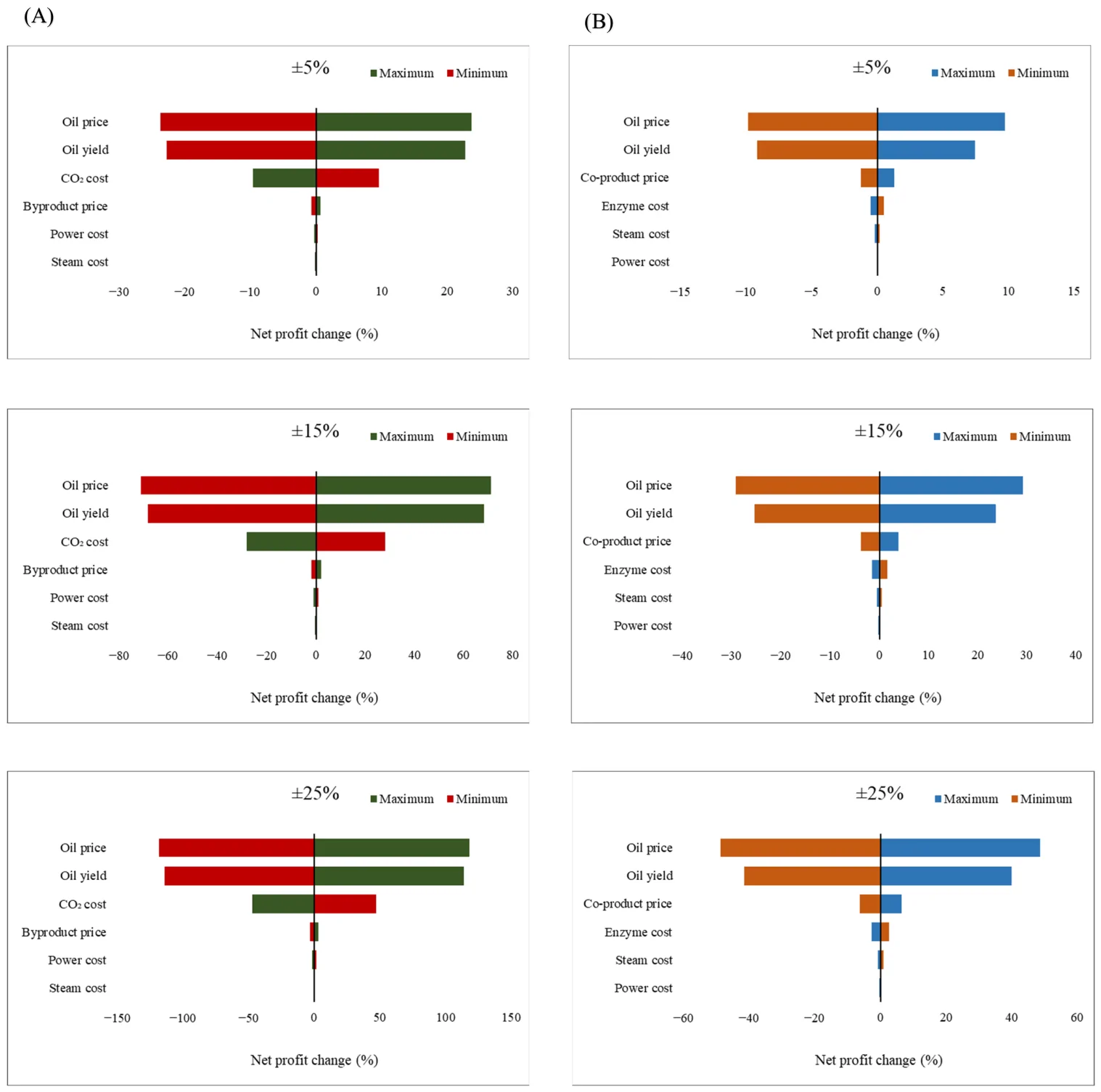
Multivariable sensitivity analysis showing the impact of key parameter variations (±5%, ±15%, and ±25%) on net profit for SCFE (**A**) and EHE (**B**). The parameters are ranked by descending influence at the ±25% variation level. For SCFE, net profit is most sensitive to the oil price, oil yield, and ${CO}_{2}$ cost. For the EHE, the oil price and oil yield dominate, with the co-product value having a moderate influence. All other parameters exhibit negligible effects.

While the throughput sensitivity analysis examined scale-up factors, the economic viability of both extraction methods is also highly dependent on market conditions and process performance parameters. To address this, a multivariable sensitivity analysis was conducted to evaluate the impact of key parameter variations (±5%, ±15%, and ±25%) on net profit for both extraction methods. The results are presented as tornado diagrams (Figure 10), with parameters ranked by descending influence at the ±25% variation level.

For SCFE (Figure 10A), net profit is most sensitive to the oil price (±118.45% change at ±25% variation), followed closely by the oil yield (±113.86%) and ${CO}_{2}$ cost (±47.36%). The dominance of the oil price and oil yield reflects their direct impact on revenue generation, while ${CO}_{2}$ cost represents the largest operating expense. The byproduct value (±3.32%), power cost (±1.51%), and steam cost (±0.76%) have negligible influences. For the EHE (Figure 10B), the oil price (±48.63%) and oil yield (±41.41%) are the most influential parameters, which is consistent with the SCFE. However, co-product value (±6.35%) has a substantially greater impact on EHE than byproduct value has on SCFE, reflecting the diversified revenue streams (cake, lecithin, soapstock) generated by the enzymatic process. The enzyme cost (±2.62%), steam cost (±0.86%), and power cost (±0.26%) have minor effects.

The analysis reveals three key insights. First, the oil price and oil yield are the dominant parameters for both technologies, indicating the importance of market conditions and extraction efficiency. Second, EHE exhibits greater overall sensitivity to revenue-side parameters (oil price, co-product value) than SCFE, reflecting its reliance on multiple value streams. Third, SCFE is more sensitive to ${CO}_{2}$ cost than EHE to any cost parameter, confirming that solvent cost management is critical for SCFE viability. From a risk perspective, the qualitative assessment indicates that SCFE is more exposed to raw material price volatility (${CO}_{2}$ cost), while EHE faces higher capital risk but benefits from revenue diversification, which provides a natural hedge against market fluctuations. These findings highlight the importance of site-specific economic conditions, particularly oil prices, co-product markets, and ${CO}_{2}$ availability, in technology selection decisions.

### 3.3. Scheduling and Visualization of the Extraction Processes

To visualize the scheduling and execution of the extraction processes, detailed equipment Gantt charts were developed for a single batch operation. In these charts, dark blue bars represent procedures, while cyan bars represent operations within those procedures. The yellow bar at the top indicates the total duration of a single batch. These charts illustrate the sequence, duration, and interdependencies of the unit operations. They provide insights into process scheduling, equipment utilization, and potential bottlenecks.

**Figure 11:**
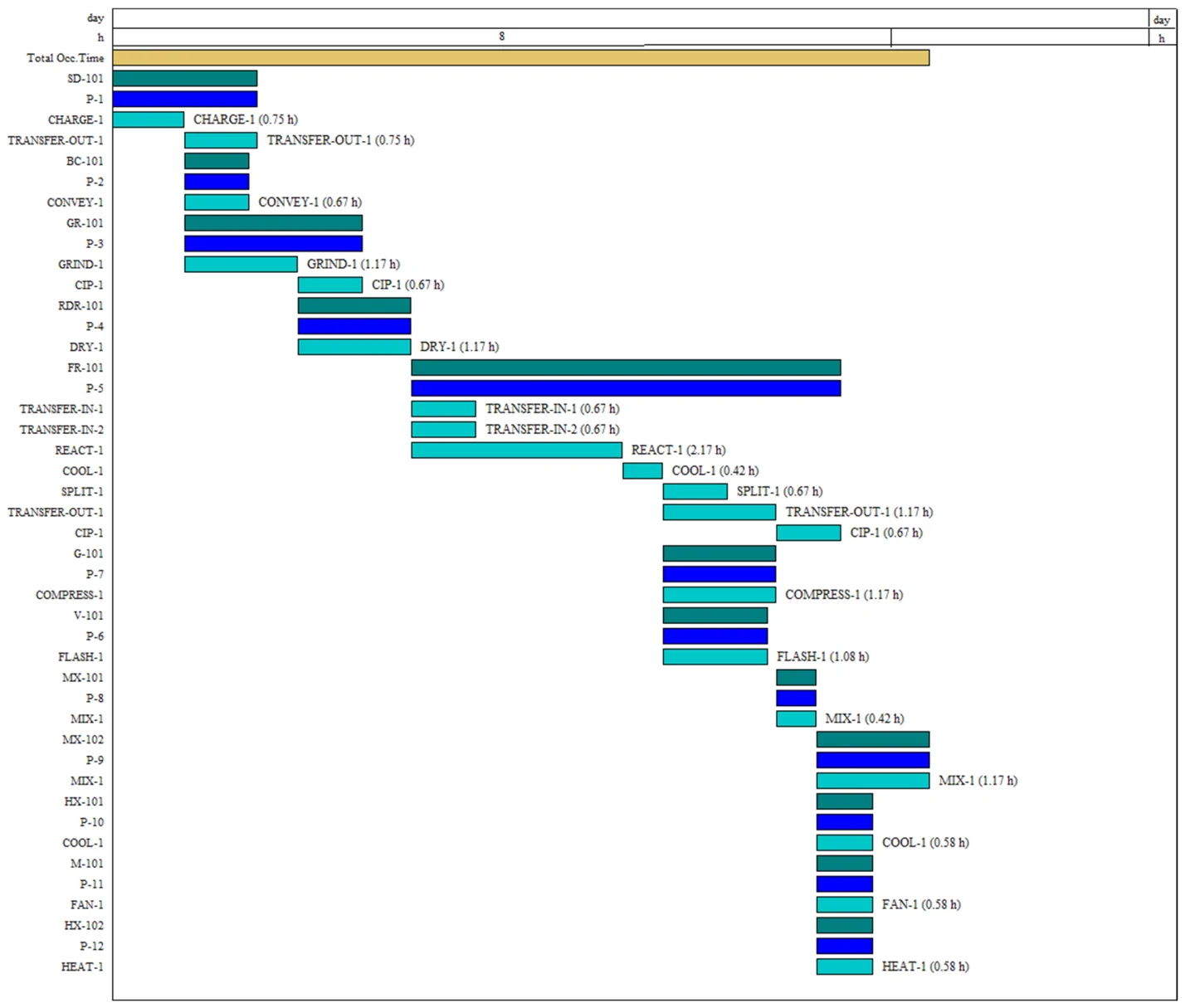
Gantt chart for the SCFE plant (single batch). The dark blue bars represent procedures, the cyan bars represent operations within those procedures, and the yellow bar at the top indicates the total batch duration (8.42 h). It illustrates the sequence and timing of unit operations, including grinding, drying, extraction, flash separation, compression, and thermal conditioning.

The SCFE process is characterized by a relatively short batch time of 8.42 h and involves 12 distinct procedures across a focused set of equipment units (Figure 11). The key operations include grinding, drying, reaction, flash separation, compression, mixing, and thermal conditioning. Notable scheduling features include a streamlined equipment footprint, employing fewer specialized units such as GR-101 (grinder), FR-101 (reactor), V-101 (flash vessel), G-101 (compressor), and RDR-101 (rotary dryer), which indicates a compact and integrated flow scheme optimized for supercritical processing. Early-stage drying integration is achieved through a rotary dryer (DRY-1 in P-6), which operates immediately after grinding to reduce the moisture content before the supercritical reaction, enhancing extraction efficiency and reducing downstream load. The process also allows for overlapping and semi-parallel operations; certain steps, such as COOL-1 and SPLIT-1 in P-3, are configured to follow previous operations without strict sequential dependency, permitting moderate reductions in equipment idle time. Pressure-driven unit operations, such as FLASH-1 and COMPRESS-1, reflect the high-pressure nature of SCFE, with compression and flash separation being critical to solvent recovery and product isolation. The Gantt chart indicates a batch time of 8.42 h, which aligns with an annual production capacity of 3583 batches, yielding 43,025,920 kg of refined fish head oil per year. This high batch frequency underscores the efficiency of the SCFE process in terms of time utilization.

**Figure 12:**
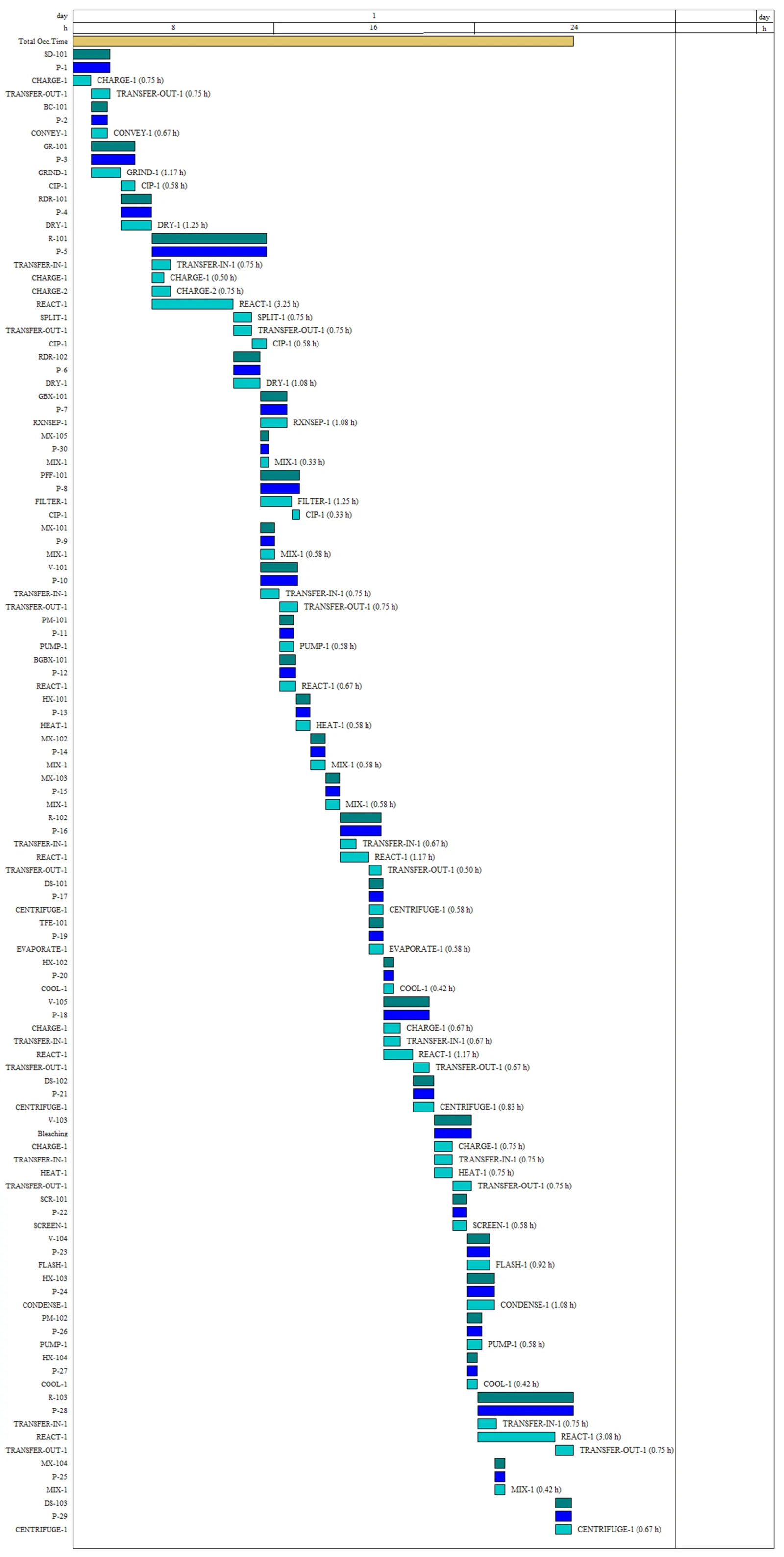
Gantt chart for the EHE plant (single batch). The dark blue bars represent procedures, cyan bars represent operations within those procedures, and the yellow bar at the top indicates the total batch duration (20 h). It shows the extended multi-stage sequence involving enzymatic hydrolysis, cold pressing, filtration, degumming, neutralization, bleaching, deodorization, and winterization.

In contrast, the enzymatic hydrolysis process requires a significantly longer batch time of 20 h and comprises around 30 procedures across a wide array of equipment, including multiple reactors, centrifuges, filters, mixers, and heat exchangers (Figure 12). Key scheduling observations include a high equipment count and procedural complexity, utilizing numerous dedicated units such as R-101, R-102, and R-103 for reactors and DS-101, DS-102, and DS-103 for centrifuges, reflecting a multi-stage extraction, purification, and polishing sequence characteristic of biocatalytic processing. Extended reaction and separation times dominate the batch timeline, with steps such as REACT-1 in P-5 and REACT-1 in P-28 each requiring 3 h, underscoring the time-sensitive nature of enzymatic reactions and subsequent separations. The process integrates multiple CIP operations across procedures, highlighting the need for stringent contamination control in food-grade and enzymatic processing. The Gantt chart data indicate a batch time of 20 h, resulting in an annual production capacity of 3448 batches, yielding 34,055,771 kg of oil per year.

The difference in the number of batches between the two extraction methods, despite their identical processing capacity, is attributed to the more efficient scheduling and reduced procedural complexity of SCFE. This method minimizes idle time between operations and enables tighter batch succession. In contrast, the EHE follows a longer, more sequential series of steps, including extended reaction, separation, and cleaning stages. These stages lengthen the critical path and limit the annual batch frequency. Both processes operate for nearly the same total annual duration (approximately 7919 h), yet SCFE completes more batches within this operational window. This demonstrates that throughput is influenced not only by batch duration but also by the efficiency of equipment sequencing and campaign design [64]. These findings underscore the importance of optimizing the procedural layout and scheduling to maximize the batch frequency in multi-stage extraction systems.

In addition to the Gantt charts, equipment occupancy charts were generated to assess the occupancy of the various equipment units for 20 batches.

**Figure 13:**
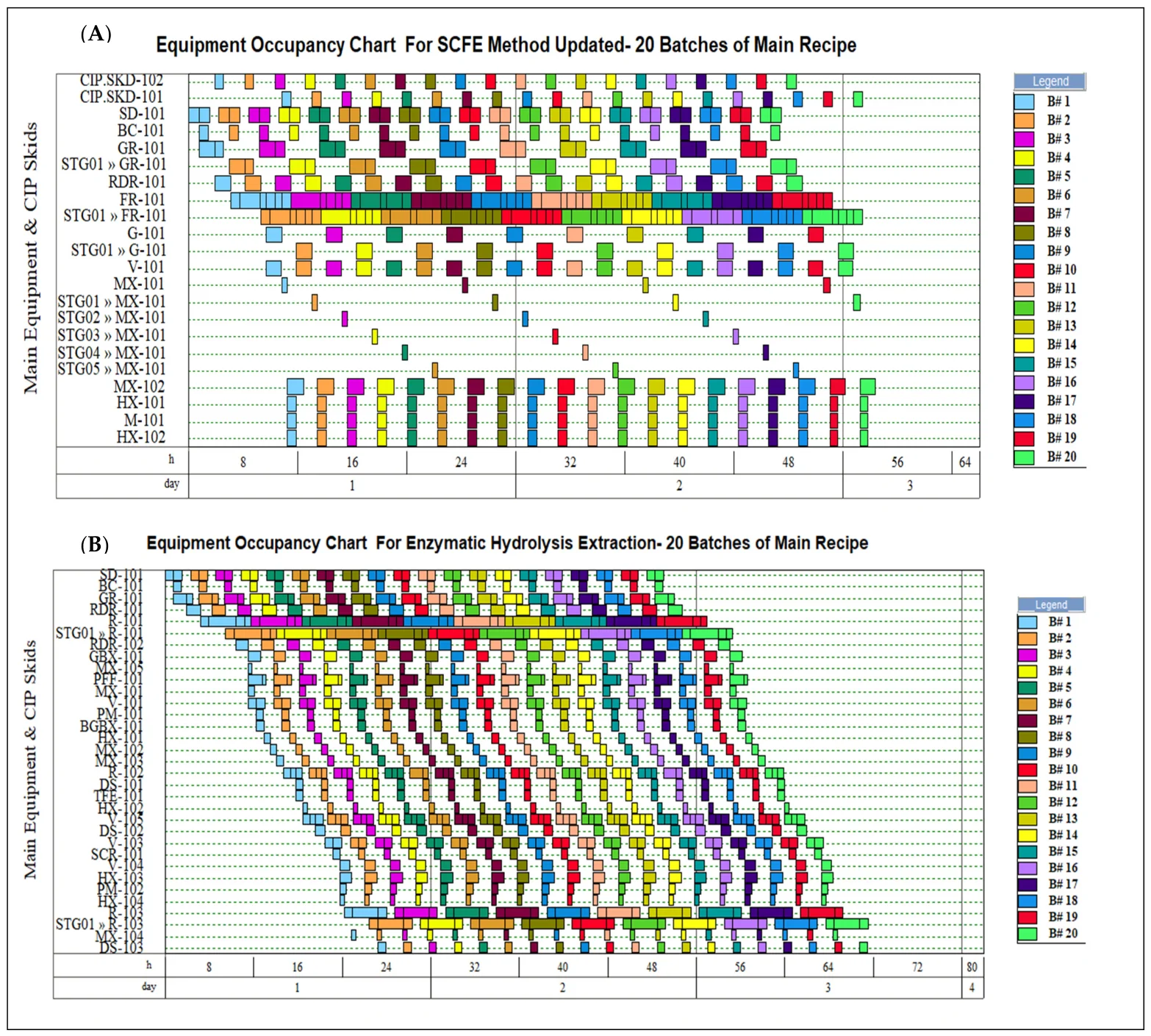
Equipment occupancy chart for 20 batches; (**A**) SCFE, (**B**) EHE.

SCFE employs parallel processing with duplicated equipment (e.g., staged reactors and grinders), resulting in a shorter total cycle time of approximately 55.5 h/20 batches (Figure 13A). Its primary bottleneck is the reactor FR-101, which operates with minimal idle time. In contrast, the EHE follows a more sequential and extended purification path, including bleaching, multiple centrifugation steps, and evaporation, leading to a total duration exceeding 63.5 h/20 batches (Figure 13B). The bottlenecks in EHE are distributed across reactors R-101/R-102 and the filter PFF-101, with noticeable idle periods in downstream equipment. Both processes efficiently integrate CIP operations directly after product transfer, reducing downtime. SCFE achieves higher throughput through parallelism, making it suitable for high-volume production.

### 3.4. Operational and Environmental Implications

#### 3.4.1. Preliminary Resource and Energy Inventory

The enzymatic hydrolysis process demonstrates superior cascading utilization of biomass, converting 60,340,000 kg/year dry fish head into multiple value streams: 34,055,771 kg/year refined oil, 28,855,713 kg/year cake, 16,037,196 kg/year lecithin, 12,390,902 kg/year soapstock, and 587,170 kg/year solid byproducts. This multi-product approach exemplifies biorefinery integration that maximizes resource efficiency from a single feedstock [65]. In contrast, SCFE processes 62,720,000 kg/year of dry fish head to yield 43,025,920 kg/year of oil alongside 31,342,080 kg/year of solid residue, which is sold as a byproduct (Table 5). Although SCFE results in lower direct water consumption than EHE (185,461,036 kg/year compared with 460,783,337 kg/year), its processing model generates a single solid residue stream rather than multiple diversified co-products. The solid residue from SCFE is modeled as a revenue-generating byproduct in the economic analysis, which is consistent with local market practices where such residues find use in animal feed or fertilizer applications. However, the single-stream output contrasts with EHE’s multiple valorization pathways, which may offer greater operational flexibility and revenue diversification. The flows presented in this section represent an operational resource inventory and qualitative discussion, not a comprehensive environmental assessment. Such an analysis is beyond the scope of the present study and is recommended as a priority for future work.

The assumption of negligible waste disposal costs in this study is context-specific. A complete waste management cost assessment would require detailed characterization of all streams, including byproducts, CIP wash water, and residual solids. However, given the efficient valorization of byproducts and the treatability of CIP waste streams through conventional wastewater systems, the process generates minimal waste relative to the large processing capacity of the plant. In this analysis, the solid residue from SCFE is sold as a byproduct ($0.50/kg) and the EHE process generates approximately fully valorized co-products, reflecting local market practices. Moreover, this assumption may vary across regions depending on local waste management infrastructure and treatment costs.

#### 3.4.2. Solvent and Chemical Use

SCFE employs 107,520,000 kg/year of supercritical ${CO}_{2}$ as its primary extraction medium. Although ${CO}_{2}$ is generally regarded as a green solvent, its environmental profile is heavily dependent on compression energy requirements at an operating pressure of 250 bar and the associated carbon footprint of compression systems. Though the system applied a ${CO}_{2}$ recovery stream, the overall analysis indicates that there is substantial net consumption. The EHE utilizes aqueous systems with 1,592,976 kg/year papain enzyme, operating at ambient pressure with significantly lower energy intensity for solvent management, indicating superior environmental performance despite higher water utilization, as water functions primarily as a reaction medium rather than a consumptive chemical.

#### 3.4.3. Energy and Carbon Flows

Direct energy consumption data indicate that SCFE requires 22,692,771 kW-h/year electricity and 211,453 metric tons/year steam, while enzymatic hydrolysis consumes 24,053,804 kW-h/year electricity and 655,868 metric tons/year steam. However, as noted in Section 3.4.1, the current analysis reports energy and material flows as an operational inventory rather than a complete environmental assessment. A comprehensive assessment would require system expansion accounting that considers the avoided burdens from displaced conventional products [65]. The EHE generates 28,855,713 kg/year high-protein cake and 16,037,196 kg/year lecithin, which are substitutes for agriculturally intensive products with substantial embedded energy and emissions. SCFE offers minimal comparable substitution potential, as its 31,342,080 kg/year solid residue needs further purification for high-value co-product recovery.

## 4. Conclusions

This study conducted a comparative TEA and process simulation for industrial-scale oil extraction from fish heads, comparing the SCFE and EHE methods using SuperPro Designer. The findings provide insights for stakeholders in the fishery and bioresource sectors considering the valorization of fish processing by-products. The simulation results, based on the assumptions and throughput range examined, reveal distinct advantages for each technology. Under the adopted assumption, SCFE results in a relatively high annual oil output (43,025,920 kg/year) and a shorter batch time (8.42 h). The throughput sensitivity analysis reveals significant diminishing returns beyond the 50,000 kg/batch design capacity, making this scale the practical economic optimum given the exponential increase in capital investment and high-pressure equipment challenges at larger sizes. Conversely, EHE, while requiring relatively higher capital investment and longer batch cycles, exhibits superior economic returns and resource efficiency under the base-case assumptions of the study. It generates multiple value-added streams, embodying an integrated biorefinery approach that maximizes biomass valorization. The process shows strong scalability, with financial metrics improving steadily up to 150,000 kg/batch, and its operating cost structure makes it less vulnerable to raw material price volatility. Therefore, the choice between SCFE and EHE should be guided by specific operational contexts. Based on the simulation framework and economic assumptions of this study, SCFE is recommended for focused, medium-scale production where solvent costs are controlled and high-purity oil is the primary target. The EHE is better suited for large-scale biorefineries aiming for maximal resource recovery, multiple revenue streams, and long-term economic resilience.

However, these recommendations should be considered in light of several key limitations. The study is simulation-based, and the integrated process models have not been experimentally validated at the pilot or industrial scale. The results are dependent on literature-derived yields and specific operations of unit procedures, fixed market prices for raw materials and products, and assumed co-product revenues. Additionally, the environmental evaluation is preliminary and does not constitute a comprehensive life-cycle assessment, and the system boundaries between the two processes are not perfectly equal, as SCFE produces a higher-purity crude oil requiring less downstream refining than EHE. Future work should prioritize experimental validation of the complete process chains, detailed characterization of oil quality from both methods, broader economic sensitivity analysis incorporating market price volatility, and a comprehensive life-cycle assessment to evaluate the full environmental impacts. Such studies will be essential to confirm the economic and sustainability benefits identified in this simulation-based assessment and to guide the sustainable scale-up of fish head valorization strategies.

## Figures and Tables

**Table 1:** Major components registered for supercritical fluid oil extraction simulation.

Component Name	Component Type	Registration Mode	Reference Component
Carb. Dioxide	Pure component	SPD Pure components data bank	${CO}_{2}$
Fish head	Stock mixture (Dry fish head 35%, water 65%)	User defined	Biomass
Oil	Pure component	User defined	Fats
Waste	Pure component	User defined	Biomass
Water	Pure component	SPD Pure components data bank	Water
Air	Stock mixture (N 76.7118%, $O_{2}$ 23.2882%)	SPD stock mixture data bank	-

**Table 2:** Major unit operations and scheduling summaries for the SCFE plant.

Procedure Name	Procedure Description	Operation	Process Scheduling	Process Description
P-1/SD-101	Storage	- Charge fish head waste → CHARGE-1. - Discharging fish head → TRANSFER-OUT-1.	- Setup time = 15 min Process time = 30 min It is the beginning of the batch. - Setup time = 15 min Process time = 30 min Starts after loading is completed.	- Loading and storage of fresh fish head waste. - Transfer to belt conveyor.
P-2/BC-101	Belt conveying	- Belt conveying → CONVEY-1.	- Setup time = 10 min Process time = 30 min Starts with TRANSFER-OUT-1.	- Start conveyor, feed fish head waste to the subsequent unit procedure (grinding), and monitor feed rate to avoid clogging.
P-3/GR-101	Grinding	- Grinding → GRIND-1 - Grinder cleaning → CIP-1	- Setup time = 10 min Process time = 60 min Starts with CONVEY-1. - Setup time = 10 min Process time = 30 min Starts after GRIND-1 ends.	- Mechanical grinding to increase surface area for extraction. - Flushing and drying grinder to eliminate residual fish particles, oils, and microbial contaminants to maintain equipment efficiency and product purity.
P-4/RDR-101	Rotary drying	- Drying → DRY-1	- Setup time = 10 min Process time = 60 min Starts after GRIND-1 ends.	- Drying to prevent ice formation during SCFE.
P-5/FR-101	Extraction	- Transfer in 1 → TRANSFER-IN-1 - Transfer in 2 → TRANSFER-IN-2 - Reaction → REACT-1 - Cooling → COOL-1 - Separation → SPLIT-1 -Transfer out → TRANSFER-OUT-1 - Reactor cleaning → CIP-1	- Setup time = 10 min Process time = 30 min Starts after DRY-1 ends. - Setup time = 10 min Process time = 30 min Starts with TRANSFER-IN-1 in this procedure. - Setup time = 10 min Process time = 120 min Starts with TRANSFER-IN-2 in this procedure. - Setup time = 5 min Process time = 20 min Starts after REACT-1 ends. - Setup time = 10 min Process time = 30 min Starts after COOL-1 ends. - Setup time = 10 min Process time = 60 min Starts with SPLIT-1. - Setup time = 10 min Process time = 30 min Starts after TRANSFER-OUT-1 ends.	- Transferring the dry contents into the extraction column. - Charging the solvent $\left({CO}_{2}\right)$. - SCFE of oil from dried fish head powder using ${CO}_{2}$. - Cool reaction contents to improve oil separation efficiency in the flash step. - Separation of the oil rich component (extract) and waste. - Discharging the waste stream. - Removes residual oil and prevents cross-contamination.
P-6/V-101	Flash	- Flash separation → FLASH-1	- Setup time = 10 min Process time = 60 min Starts with SPLIT-1.	- Flash separation of oil from ${CO}_{2}$ via depressurization.
P-7/G-101	Gass compression	- Centrifugal gas compression → COMPRESS-1	- Setup time = 10 min Process time = 60 min Starts with FLASH-1	- Recompresses ${CO}_{2}$ for reuse in the extraction loop.
P-8/MX-101	Mixing	- Mix → MIX-1	- Setup time = 5 min Process time = 20 min Starts with TRANSFER-IN-2 of extraction procedure.	- Mixing the supply and recycled ${CO}_{2}$.
P-9/MX-102	Mixing	- Mix → MIX-1 (custom mixing)	- Setup time = 10 min Process time = 60 min Starts after MIX-1 operation in previous procedure ends.	- Mixing the make-up and recycled ${CO}_{2}$ and adjusting the flow rate.
P-10/HX-101	Cooling	- Gas cooling → COOL-1	- Setup time = 5 min Process time = 30 min Starts with MIX-1 operation in previous procedure.	- Cools and liquefies ${CO}_{2}$.
P-11/M-101	Gas flow	- Centrifugal fan → FAN-1	- Setup time = 5 min Process time = 30 min Starts with COOL-1 operation in cooling procedure.	- Transport ${CO}_{2}$ into heat exchanger.
P-12/HX-102	Heating	- Heat → HEAT-1	- Setup time = 5 min Process time = 30 min Starts with FAN-1.	- Heats ${CO}_{2}$ to supercritical state for extraction.

**Table 3:** Components registered for simulations of enzymatic hydrolysis-based oil extraction.

Component Name	Component Type	Registration Mode	Reference Component
Air	Stock mixture (N = 76.7118%, $O_{2}$ = 23.2882%)	SPD stock mixture data bank	-
Bleaching Earth	Pure component	User defined	-
Cake	Pure component	User defined	Biomass
Color	Pure component	User defined	-
Crude oil	Pure component	User defined	Oil
Enzyme (Papain)	Pure component	User defined	-
FFA	Pure component	User defined	Fats
Fish head	Stock mixture (Dry fish head = 35%, water = 65%)	User defined	Biomass
Glycerol	Pure component	User defined	Fats
Hot air	Pure component	User defined	Air
Lecithin	Pure component	User defined	Fats
Linoleic acid	Pure component	SPD Pure components data bank	Linoleic acid
Nitrogen	Pure component	SPD Pure components data bank	Nitrogen
Oleic acid	Pure component	SPD Pure components data bank	Oleic acid
Oxygen	Pure component	SPD Pure components data bank	Oxygen
Phospholipids	Pure component	User defined	Fats
Phospholipids solid	Pure component	User defined	Fats
Phosphoric acid	Pure component	User defined	Water
Precipitated/wax	Pure component	User defined	Fats
Soaps	Pure component	User defined	Fats
NaOH	Pure component	User defined	NaOH
Stearic acid	Pure component	SPD Pure components data bank	Stearic acid
TAG-Polyunsaturated	Pure component	User defined	Oil
TAG-Saturated	Pure component	User defined	Oil
TAG-Unsaturated	Pure component	User defined	Oil
Triglycerides	Pure component	User defined	Fats
Volatiles	Pure component	User defined	-
Water	Pure component	SPD Pure components data bank	Water
Wax	Pure component	User defined	Fats
NaOH (20% *w*/*w*)	Stock mixture	SPD stock mixtures data bank	Water

TAG = Tri-Acyl-Glycerides.

**Table 4:** Major unit operations and scheduling summaries for the EHE plant.

Procedure Name	Procedure Description	Operation	Process Scheduling	Process Description
P-1/SD-101	Storage	Charge fish head waste → CHARGE-1 Discharging fish head → TRANSFER OUT-1	- Setup time = 15 min Process time = 30 min Beginning of the batch - Setup time = 15 min Process time = 30 min Starts when CHARGE-1 operation in this procedure ends.	- Loading and storage of raw material.
P-2/BC-101	Belt conveying	Moving fish head → CONVEY-1	- Setup time = 15 min Process time = 30 min Starts with TRANSFER OUT-1 operation in previous procedure.	- Transferring fish head from storage tank to grinding section.
P-3/GR-101	Grinding	Grind fish heads to paste → GRIND-1 Grinder cleaning → CIP-1	- Setup time = 10 min Process time = 60 min Starts with CONVEY-1 operation. Setup time = 5 min Process time = 30 min Starts after GRIND-1 ends.	- Particle size reduction to enhance enzyme contact. - Removes residual tissue and prevents microbial growth.
P-4/RDR-101	Rotary drying	Drying fish head paste → DRY-1	- Setup time = 15 min Process time = 60 min Starts after GRIND-1 operation ends.	- Removes moisture content and create a stable, standardized raw material.
P-5/R-101	Extraction	Transfer hydrolysate → TRANSFER-IN-1 Charge enzyme → CHARGE-1 Charge water → CHARGE-2 Reaction → REACT-1 Reaction component splitting → SPLIT-1 Discharging oil → TRANSFER-OUT-1 Reactor cleaning → CIP-1	- Setup time = 15 min Process time = 30 min Starts after DRY-1 operation ends. Setup time = 10 min Process time = 20 min Starts with TRANSFER-IN-1 operation. - Setup time = 15 min Process time = 30 min Starts with TRANSFER-IN-1 operation. - Setup time = 15 min Process time = 180 min Starts with TRANSFER-IN-1 operation. - Setup time = 15 min Process time = 30 min Starts after REACT-1 operation ends. - Setup time = 15 min Process time = 30 min Starts after REACT-1 ends. - Setup time = 5 min Process time = 30 min -Starts after TRANSFER-OUT-1 operation ends.	- Mixing ground paste with water and specific enzyme (papain). - Controlled reaction to break down proteins and release oil. - Oil and cake separation. - Removing extracted oil from reactor. - Removes reaction residual, prevents cross-contamination, and ensure the hygienic safety of subsequent batches.
P-6/RDR-102	Rotary drying	Remove reaction water → DRY-1	- Setup time = 5 min Process time = 60 min Starts with TRANSFER-OUT-1 operation in previous procedure.	- Reduce water content and make cake suitable for subsequent extraction.
P-7/GBX-101	Cold pressing	Second stage extraction → RXNSEP-1	- Setup time = 5 min Process time = 60 min Starts after DRY-1 operation ends.	- Mechanical pressing of the residual cake to recover additional bound oil. - Maximizes overall oil yield from the process.
P-30/MX-105	Mixing	Mixing oil recovered → MIX-1	- Setup time = 5 min Process time = 15 min Starts with RXNSEP-1 operation.	- Mixing oil recovered directly from hydrolysis and subsequent cold pressing.
P-8/PFF-101	Filtration	Component separations → FILTER-1 Filter cleaning → CIP-1	- Setup time = 15 min Process time = 60 min Starts with MIX-1 operation in previous procedure. - Setup time = 5 min Process time = 15 min Starts after FILTER-1 operation ends.	- Separation of crude fish oil and fine solids from both hydrolysis and pressing procedures. - Flushing plate and frame filter to avoid blockage and improve efficiencies.
P-9/MX-101	Mixing	Mixing solid residue → MIX-1	- Setup time = 5 min Process time = 30 min Starts with FILTER-1 operation in previous procedure.	- Mixing fine solids and initial cake from extraction chamber, and store it as co-product.
P-10/V-101	Blending/storage	Store crude oil → TRANSFER-IN-1 Discharge crude oil into next procedure → TRANSFER-OUT-1	- Setup time = 15 min Process time = 30 min Starts with FILTER-1 operation in previous procedure. - Setup time = 15 min Process time = 30 min Starts after TRANSFER-IN-1 operation in this procedure ends.	- Collect crude oil from filtration procedure. - Transferring crude oil into subsequent procedures.
P-11/PM-101	Oil pumping	Pumping crude oil → PUMP-1	- Setup time = 15 min Process time = 30 min Starts with TRANSFER-OUT-1 operation in previous procedure.	- Transferring oil into main refining section.
P-12/BGBX-101	Generic procedure	Crude oil conversion to its components → REACT-1	- Setup time = 10 min Process time = 30 min Starts with PUMP-1 operation.	- Involves a reaction that converts the crude oil to its basic components.
P-13/HX-101	Heating	Heating → HEAT-1	- Setup time = 5 min Process time = 30 min Starts after REACT-1 operation ends.	- Enhance the efficiency of removing non-hydratable phosphatides.
P-14/MX-102	Mixing	Mixing streams → MIX-1	- Setup time = 5 min Process time = 30 min Starts after HEAT-1 operation in previous procedure ends.	- Adding phosphoric acid and mixing for effective degumming of the crude oil.
P-15/MX-103	Mixing	Adding water to previous mixture → MIX-1	- Setup time = 5 min Process time = 30 min Starts after MIX-1 operation in previous procedure ends.	- It hydrates and remove phospholipids (gums).
P-16/R-102	Phospholipids hydration	Transfer mixture to reaction chamber → TRANSFER-IN-1 Reaction → REACT-1 Discharge reaction products → TRANSFER-OUT-1.	- Setup time = 10 min Process time = 30 min Starts after MIX-1 operation in previous procedure ends. - Setup time = 10 min Process time = 60 min Starts after MIX-1 operation in previous procedure ends. - Setup time = 10 min Process time = 30 min Starts after REACT-1 operation in this procedure ends.	- Transfer of the crude oil containing phosphoric acid and degumming water into reaction chamber. - Solidifying phospholipids (gums) out of the oil phase. - Transferring reaction contents into centrifuge to remove solid phospholipids.
P-17/DS-101	Degumming	Centrifugation → CENTRIFUGE-1	- Setup time = 5 min Process time = 30 min Starts after TRANSFER-OUT-1 operation in previous procedure ends.	- Removal of solid phospholipids using centrifuge.
P-18/V-105	Neutralization	Feed NaOH → CHARGE-1 Transfer non solid oil components → TRANSFER-IN-1 Saponification → REACT-1 Discharging into subsequent procedure → TRANSFER-OUT-1	- Setup time = 10 min Process time = 30 min Starts after CENTRIFUGE-1 operation in previous procedure ends. - Setup time = 10 min Process time = 30 min Starts with CHARGE-1 operation in this procedure. - Setup time = 10 min Process time = 60 min Starts with TRANSFER-IN-1 operation in this procedure. - Setup time = 10 min Process time = 30 min Starts after REACT-1 operation in this procedure.	- Addition of NaOH to remove Free Fatty Acids (FFAs) by converting them into water-soluble soap (soapstock). - Transfer non solid oil components from centrifuge to the reaction chamber. - Saponification of free fatty acids using NaOH solution. - Discharging the neutralized oil mixture from the reactor to the subsequent procedure.
P-19/TFE-101	Evaporation	Dewatering gums → EVAPORATE-1	- Setup time = 5 min Process time = 30 min Starts with CENTRIFUGE-1 operation in P-17/DS-101 procedure.	- Gum solids from P-17/DS-101 are dewatered.
P-20/HX-102	Cooling	Cooling to room temperature → COOL-1	- Setup time = 5 min Process time = 20 min Starts after EVAPORATE-1 operation in previous procedure.	- Dewatered gums are cooled to room temperature and packed as lecithin.
P-21/DS-102	Centrifugation	Soap removal → CENTRIFUGE-1	- Setup time = 5 min Process time = 45 min Starts with TRANSFER-OUT-1 operation in procedure P-18/V-105.	- Centrifugation to remove soaps.
Bleaching/V-103	Bleaching	Addition of bleaching earth → CHARGE-1 Transferring oil from centrifuge → TRANSFER-IN-1 Heating → HEAT-1 Discharging contents → TRANSFER-OUT-1	- Setup time = 15 min Process time = 30 min Starts with TRANSFER-IN-1 operation in this procedure. - Setup time = 15 min Process time = 30 min Starts after CENTRIFUGE-1 operation in P-21/DS-102 procedure ends. Setup time = 10 min Process time = 30 min Starts with TRANSFER-IN-1 operation in this procedure. - Setup time = 15 min Process time = 30 min Starts after HEAT-1 operation in this procedure ends.	- Addition of bleaching earth to remove color agents. - Transfering oil to the bleaching vessel for color removal. - Heating to reduce viscosity, which improves the dispersion of the bleaching earth, and to maximize the adsorption of pigments (e.g., carotenoids, chlorophyll). - Discharging the bleached oil mixture from the bleaching vessel to the next unit operation.
P-22/SCR-101	Stationary screening	Bleaching earth removal → SCREEN-1	- Setup time = 5 min Process time = 30 min Starts with TRANSFER-OUT-1 operation in previous procedure.	- Removing the bleaching earth and impurities using a stationary screen.
P-23/V-104	Deodorization	Volatile removal → FLASH-1	- Setup time = 10 min Process time = 45 min Starts after SCREEN-1 operation in previous procedure ends.	- Flash separation of volatile components of oil.
P-24/HX-103	Condensation	Vapor condensation → CONDENSE-1	- Setup time = 5 min Process time = 60 min Starts with FLASH-1 operation in previous procedure.	- Vapor condensation and transfer to soap stock mixture.
P-25/HX-103	Mixing	Soap mixing → MIXING-1	- Setup time = 5 min Process time = 20 min Starts after CONDENSE-1 operation in previous procedure ends.	- Underflow stream from P-21/DS-102 and condensed vapor from P-24/HX-103 procedures are mixed and packed as by product (soap-stock).
P-26/PM-102	Fluid flow	Pumping oil → PUMP-1	- Setup time = 5 min Process time = 30 min Starts with FLASH-1 operation in P-23/V-104 procedure.	- Pumping oil to cooling section.
P-27/HX-104	Cooling	Cooling → COOL-1	- Setup time = 5 min Process time = 20 min Starts with FLASH-1 operation in P-23/V-104 procedure.	- Reduce oil temperature from 210 °C (flash operating temperature) into around 30 °C.
P-28/R-103	Winterization	Oil reception → TRANSFER-IN-1 Reaction → REACT-1 Discharging oil from reactor → TRANSFER-OUT-1	- Setup time = 5 min Process time = 30 min Starts after COOL-1 operation in previous procedure ends. - Setup time = 5 min Process time = 180 min Starts with TRANSFER-IN-1 operation in this procedure. - Setup time = 15 min Process time = 30 min Starts after REACT-1 operation in this procedure ends.	- Reception of oil from cooling procedure for further purification. - Conversion of waxes into precipitates under low temperature (12 °C). - Discharging the winterized oil from the reactor to the subsequent centrifugation unit.
P-29/DS-103	Centrifugation	Precipitated wax removal → CENTRIFUGE-1	- Setup time = 10 min Process time = 30 min Starts with TRANSFER-OUT-1 operation in previous procedure.	- A final stage that remove precipitated waxes using centrifugation and discharge the refined oil as overflow.

**Table 5:** Major economic model assumptions for both extraction methods.

Method	Item	Purchase Price ($/)	Selling Price ($/)	Reference
SCFE	Fish head waste	0.5/kg	-	[49]
	${CO}_{2}$	4.15/kg	-	[50]
	Water	0.15/kg	-	[51]
	Refined Fish Head Oil (Main Product)	-	13.00/kg	[52]
	Waste (Byproduct)	-	0.50/kg	[49]
	Steam (Utility)	12/MT	-	[53]
	Std Power (Utility)	0.1/kW-h	-	[53]
EHE	Fish head waste	0.5/kg	-	[49]
	Enzyme (Papain)	15/kg	-	[54]
	Water	0.15/kg	-	[55]
	Refined Fish Head Oil (Main)	-	13/kg	[52]
	Cake (Byproduct)	-	1.00/kg	[49]
	Lecithin (Byproduct)	-	1.00/kg	[49]
	SoapStock (Byproduct)	-	1.00/kg	[49]
	Solid Byproducts	-	1/kg	[49]
	Steam (Utility)	12/MT	-	[53]
	Std Power (Utility)	0.1/kW-h	-	[53]

Std Power, Standard Power; MT, Metric Ton.

**Table 6:** Mass balance summary for the SCFE and EHE processes.

Parameter	SCFE	EHE
Inputs		
Raw fish head feed (kg/batch)	50,000	50,000
Oil content in feed (kg/batch)	12,500	12,500
Circulating ${CO}_{2}$ (kg/batch)	300,000	-
Make-up ${CO}_{2}$ (kg/batch)	14,900	-
Water (kg/batch)	125,849	133,638
Enzyme (kg/batch)	-	462
Hot air (kg/batch)	146,250	474,804
Other chemicals (kg/batch)	-	2934
Outputs		
Refined oil (kg/batch)	12,005	9877
Oil recovery efficiency (%)	96.04	79.02
Cake (kg/batch)	-	6131
Lecithin (kg/batch)	-	4695
Soapstock (kg/batch)	-	3594
Solid byproducts (kg/batch)	8745	170
Water vapor removed during drying (kg/batch)	29,250	474,804
${CO}_{2}$ recycled to next batch (kg/batch)	285,000	-
${CO}_{2}$ lost/vented (kg/batch)	15,000	-

**Table 7:** Process efficiency and material utilization comparison.

Parameter	SCFE	EHE
Annual oil output (MP)	43,025,920.00 kg MP/yr	34,055,771 kg MP/yr
Oil recovery (% of feed mass)	24.01	19.75
Oil recovery efficiency (% of oil content)	96.04	79.02
Circulating ${CO}_{2}$ (kg/batch)	300,000	-
Circulating ${CO}_{2}$ (kg/kg oil)	25	-
Make-up ${CO}_{2}$ (kg/batch)	14,900	-
Make-up ${CO}_{2}$ (kg/kg oil)	1.241	-
Water consumption (kg/kg oil)	10.48	13.53
Enzyme consumption (kg/kg oil)	-	0.047
Hot air consumption (kg/kg oil)	12.18	48.07
Utility cost ($/year)	5,100,000	11,000,000
Material recovery	High-purity oil, low byproduct generation.	Multiple byproducts (cake, lecithin, soapstock, etc.)
Energy intensity	Lower utility cost ($5.1 M/yr).	Higher utility cost ($11 M/yr) due to steam and heating.
Waste generation	Low	Minimal

Waste disposal cost was considered negligible for both extraction methods.

**Table 8:** Summary of the main techno-economic metrics for both extraction methods.

Item	Metrices	SCFE	EHE
Economics	Total investment ($)	846,214,000	862,731,450
	Total revenue ($)	574,848,000	500,596,008
	Operating cost ($/year)	456,872,000	272,840,215
	Unit Production Ref. Rate (kg MP/yr)	43,025,920	34,055,771
	Unit Production Cost ($/kg MP)	10.62	8.0116
	Unit Production Revenue ($/kg MP)	13.36	14.6993
Project indices	Gross Margin (%)	20.52	45.50
	ROI (%)	17.11	24.77
	Payback Time (Years)	5.84	4.04
	IRR Before Taxes (%)	11.02	26.64
	IRR After Taxes (%)	11.02	18.36
	NPV at (7.00%) ($)	210,911,000	673,903,143
Size	Batch Throughput (kg MP/batch)	12,005.00	9876.96
	Annual Throughput (kg MP/yr)	43,025,920.00	34,055,771.25

IRR: internal rate of return; NPV: net present value; MP: total flow of stream ‘refined fish head oil’.

## Data Availability

The original contributions presented in this study are included in the article. Further inquiries can be directed to the corresponding authors.
